# The importance of low IQ to early diagnosis of autism

**DOI:** 10.1002/aur.2842

**Published:** 2022-11-13

**Authors:** Kristina Denisova, Zhichun Lin

**Affiliations:** ^1^ Laboratory of Autism Origins and Mind and Brain Development, Division of Math and Natural Sciences, Department of Psychology, Queens College and Graduate Center City University of New York New York New York USA

**Keywords:** autism spectrum disorder, early childhood autism, infants, intelligence quotient, prospective study

## Abstract

Some individuals can flexibly adapt to life's changing demands while others, in particular those with Autism Spectrum Disorder (ASD), find it challenging. The origin of early individual differences in cognitive abilities, the putative tools with which to navigate novel information in life, including in infants later diagnosed with ASD remains unexplored. Moreover, the role of intelligence quotient (IQ) vis‐à‐vis core features of autism remains debated. We systematically investigate the contribution of early IQ in future autism outcomes in an extremely large, population‐based study of 8000 newborns, infants, and toddlers from the US between 2 and 68 months with over 15,000 cross‐sectional and longitudinal assessments, and for whom autism outcomes are ascertained or ruled out by about 2–4 years. This population is representative of subjects involved in the National Institutes of Health (NIH)‐funded research, mainly on atypical development, in the US. Analyses using predetermined age bins showed that IQ scores are consistently lower in ASD relative to typically developing (TD) children at all ages (*p* < 0.001), and IQ significantly correlates with social, non‐social, and total Calibrated Severity Scores (CSS) on the Autism Diagnostic Observation Schedule (ADOS) (*p*<0.01). Lower IQ is associated with greater autistic impairments. Note, verbal IQ (VIQ) is no better than the full‐scale IQ to predict ASD cases. These findings raise new, compelling questions about potential atypical brain circuitry affecting performance in both verbal and nonverbal abilities and preceding an ASD diagnosis. This study is the first to establish prospectively that low early IQ is a major feature of ASD in early childhood.

**Lay Summary:**

The role of IQ scores in autism remains debated. We systematically investigate the contribution of early IQ in an extremely large study of 8,000 children between 2 and 68 months with autism outcomes by about 2–4 years. We show that IQ scores are consistently lower in ASD relative to TD children. This study is the first to establish prospectively that low early IQ is a predictor for ASD diagnosis in early childhood.

## INTRODUCTION

Intellectual ability is currently conceptualized as a distinct dimension relative to the core features of Autism Spectrum Disorder (ASD). However, epidemiological data from different countries indicate a high prevalence of low intelligence quotient (IQ) scores in children with ASD. According to the most recent ASD surveillance estimates in the US, 35% of 8‐year‐old children with ASD have Intellectual Disability (ID) (IQ < 70, Maenner et al., 2021). Similarly, an epidemiological study of 7–12‐year‐old children in South Korea reveals that about 1/3 of children with ASD have IQ less than 70 (33% in the high probability group and 25.9% from the general population, Kim et al., 2011). A UK report on a set of children (*N* = 156, 10–14 years) seen as part of an epidemiological Special Needs and Autism Project (SNAP) finds that as much as 55% of the children with ASD have an intellectual disability (IQ < 70) (Charman et al., 2011).

It is still open to question whether lower IQ might be a secondary effect of severe autism symptoms, or whether on the contrary it might causally affect the severity of the symptoms and if so, for which subset of individuals with ASD. Early case studies hint at the contribution of higher IQ scores to future social adjustment outcomes in children with autism (Rutter et al., 1967, p. 11), with low IQ indicating a poor autism prognosis (Carr, 1976). Higher IQ in preschool as well as communicative speech before 6 years of age was found to be associated with better outcomes and prognosis for children with autism (e.g., Gillberg & Steffenburg, 1987). A recent literature review indicates that positive outcomes are more likely for “individuals with higher childhood IQs and language development by the age of 5–6 years” (Levy & Perry, 2011).

The association between autism, IQ, and adaptive functioning has been probed in recent epidemiological and prospective cohort studies. (Impairment in everyday adaptive functioning is one of the essential criteria for diagnosing ID in DSM‐5 (American Psychiatric Association, 2013), p. 37). Children with ASD drawn from the general population have lower adaptive skills relative to their IQ scores; moreover, lower adaptive scores associate with magnitude of “early social impairment” (Charman et al., 2011). Studies with infants at a high familial, genetic risk (HR) for ASD (due to an older sibling diagnosed with ASD) indicate that lower IQ is associated with lower adaptive functioning (Bussu et al., 2019; Zwaigenbaum et al., 2021). Further, a study by Salamone and colleagues (2018) examining subdomain scores on the Mullen starting with 7 months and up to a 7 years at follow‐up in the BASIS sample, reveals consistently lower scores for HR relative to LR infants, with increasing difficulties in adaptive behaviors detected in HR‐ASD‐positive, but not HR‐ASD‐negative or LR children (Salomone et al., 2018).

Intellectual capacities and skills that support active acquisition of information and enable efficient learning by the developing brain “on the fly” may contribute to both higher IQ scores and lower ASD symptoms (and/or normative social functioning and social communication). Conversely, a number of studies (e.g., Denisova, 2019; Denisova & Zhao, 2017; Jones & Klin, 2013), in particular, of HR infants for ASD, have explored possible disruption of basic social and/or attentional mechanisms, which may deleteriously impact intellectual functioning.

Earlier researchers were particularly interested in the effect of atypical acquisition of language in children with autism. They argued that impaired language processing ability might restrict normal socialization and social maturity (Lockyer & Rutter, 1970). Indeed, language “plays an important part in the growth of intelligence” (Lockyer & Rutter, 1970). During the first year of life, innate competencies provide scaffolding during language learning and acquisition (e.g., Saffran et al., 1996). Processes underlying language delay in children, such as those who are ‘late talkers’, are likely distinct from processes underpinning atypical communication, both verbal and non‐verbal. In early life, cognitive processes may help shape the processing of initially undifferentiated information in the ambient environment, for example, in the auditory stream, and help “attune” the developing brain to features specific to one's native language (Denisova, 2019). One possibility is that the robustness of this tuning process may either impede or facilitate development of communication skills. Recent data indicate that initial speech delay in young children may herald additional problems, including global delay (e.g., Zengin‐Akkuş et al., 2018), and suggests a potential overlap between language delay and autism (e.g., Jiménez et al., 2021), including in adults with autism (e.g., Armstrong et al., 2017).

Individual differences in early cognitive abilities, the putative tools with which to navigate novel information in life, to adapt flexibly in changing environments, and to judge well (Binet & Simon, 1916), remain unexplored for their potential role as a major contributor to core social and non‐social symptoms of ASD, in particular pertaining to impairments in the theory of mind (Velikonja et al., 2019). Relatedly, not only ASD children show the greatest impairments on socio‐cognitive tasks, considered precursors to atypical ability to think about others' minds (Frith, 1989), ASD children also show delays on tasks requiring cooperation with other people (Ellis et al., 2020). Atypical information processing has been consistently detected in children and adults with ASD (e.g., using functional Magnetic Resonance Imaging [MRI] and a cognitive interference task; Denisova et al., 2013, 2016). Potential atypicalities in information processing in *early childhood* may interfere with ability to adjust thinking to new conditions in life (Stern, 1914).

Given that on average diagnosis is relatively late with around 4–5 years of age (e.g., 51 months [Maenner et al., 2021]), identifying early signs of ASD is important for discovering early mechanisms potentially driven by IQ differences. This average might subsume a wide range and if so, hint at two or more populations: some individuals who are diagnosed relatively early (severe ASD symptoms, lower IQ) and some relatively late (milder symptoms, higher IQ).

Improved understanding of the role of *early* IQ in autism manifestations would allow the possibility to re‐conceptualize how mental and neural development unfolds in some infants. This important aim is challenging, as reflected by the complicated requirement for ascertaining comorbid ASD and ID in clinical practice, such that social communication is lower than what would be expected for “general developmental level” (DSM‐5 [American Psychiatric Association, 2013, p. 51]). Further, DSM‐5 advises delaying diagnoses of ID in young infants and toddlers, prior to a course of intervention (DSM‐5 [American Psychiatric Association, 2013, p. 39]). The relative uncoupling of intelligence levels from diagnosis makes it more challenging to generate research‐driven insights on early drivers of ASD as a function of IQ differences in very young children.

A recent HR infant siblings study reported cases with missed early diagnoses around 3 years of age (Ozonoff et al., 2018) of children who were not considered to have ASD at that early time point, but who were later ascertained to have ASD. Importantly, these children later diagnosed with ASD had normal or average IQ (Ozonoff et al., 2018). That is, the cases that were missed during earlier assessments were *not* characterized by early low IQ. This finding again implicates the important role of early low (but not high) IQ for stable early ASD diagnoses, at least in the high risk population.

Encouragingly, in an early study, when corrected for the reliability of the test (0.90) (given a correlation of about 0.30, observed between 6 months and 3 years; Hindley & Owen, 1978), only relatively small changes are to be expected in the median at the second testing. To increase confidence in the results of this study of cognitive ability estimates at early ages, we interrogate the reliability of early IQ estimates directly with the data at hand. Moreover, whether IQ is reliable in children with ASD *per se* is not fully clear, but as reported in the HR study above, stable ASD diagnoses are characterized by early low IQ (Ozonoff et al., 2018). Conversely, an important question is whether IQ is reliable when estimated at extremely early ages.

Only a handful of studies examined IQ relative to the age of ASD onset. Low cognitive abilities are detected in children from the general population who are diagnosed with ASD relatively early, under 2 years of age (e.g., Chawarska et al., 2007, cf. Shumway & Wetherby, 2009). Lower IQ is detected in ASD relative to typically developing (TD) individuals in a sample with a wide range of age at first visit (~3 to 39 years of age, initial non‐verbal IQ > 70), with the differences diminishing in an adults‐only (18 years and above at first visit) between‐group comparison (Prigge et al., 2021). This analysis suggests that participants below 18 years are contributing to the overall finding of lower IQ. In a sample with an initial age at first visit around 28–69 months (and no group matching on IQ), the full‐scale IQ (ELC) on the Mullen is lower in ASD vs. TD individuals (Girard et al., 2021). Given the evidence of lower IQ in children diagnosed with ASD in early childhood, we may further expect early low IQ scores to associate with worse ASD manifestations in children diagnosed relatively early in childhood.

### Current study

Our key hypotheses focus on the relationship of early IQ data to later ASD manifestations in the same children, ascertained using the Autism Diagnostic Observation Schedule (ADOS) and DSM‐5 criteria. In order to probe this relationship for as many children as possible using the same instrument (Mullen Scales of Early Learning, MSEL [Mullen, 1995]), we leverage the structure and mission of the NIMH Data Archive (NDA). In total, the 8000 subjects in the original studies represent those involved in research currently funded by the National Institutes of Health (NIH) in the US, as all NIH‐funded researchers are required to submit their data acquired with human subjects to the NDA. These children are representative of participants in study populations recruited primarily into ASD research studies in the US. For this study, the key inclusion criterion for all children was the availability of valid administration on the MSEL (Mullen, 1995), which yielded a set of over 8000 infants and toddlers (including 1956 tested longitudinally). Within that set, subjects had to have been administered the ADOS, with the outcome status of ASD or TD ascertained by a research‐reliable team member on the original studies using the ADOS and expert clinical opinion (a third group for which ASD was ruled out, but TD status not conferred, was also examined in supplementary analyses).

We further investigate how the severity of social and non‐social autism manifestations (on the ADOS, using the Calibrated Severity Scores) relates to IQ. Crucially, we pursue the hypothesis that early low IQ is a risk factor for ASD diagnosis by around 3–4 years. Exploratory analyses investigated related questions, with arising important questions noted for future work.

## METHODS

### Datasets and information on the recruitment of subjects into studies in the NIMH Data Archive (NDA)

Data used in the preparation of this study were obtained from the United States National Institutes of Health (NIH)‐supported National Database for Autism Research (NDAR). NDAR is a collaborative informatics system created by the National Institutes of Health to provide a national resource to support and accelerate research in autism. NDAR is now part of the NDA. Importantly, all investigators currently funded by the NIH and conducting human subjects research are required to submit original, raw data collected from NIH‐supported studies directly into the NDA. As such, researchers submitting data to the Archive pursue different scientific questions about mind, brain, and behavior in health and illness. The subjects include children who may be atypically developing (or those at risk for atypical development) as well as typically developing children. Note that the typically developing children may be recruited as controls in studies of atypically developing children, or they may be recruited into studies of normal development (such as infants recruited into studies of twins); these children are not expected to have a priori risks for developmental problems.

For the purpose of this current study, focusing on characterization of early cognitive abilities in children on the MSEL (Mullen, 1995), we developed strict inclusion/exclusion criteria, which we applied at the level of each individual subject's dataset, not at the level of a given study‐site or geographic location. This multi‐pronged process included stringent quality checks applied to each individual dataset, imposing a uniform data quality control and permitting thorough characterization of the data, to produce the largest dataset possible with which to investigate early cognitive abilities on MSEL (Mullen, 1995), from a total of 62 study‐sites.

Data initially were not excluded on the basis of subject or family characteristics related to potential medical or genetic conditions associated with ASD or related atypical development; there are three reasons for this. First, when recruitment and subject information is collected in a uniform manner in a single autism laboratory, it is possible to exert stringent and precise control over specific exclusion criteria, including medical conditions known to associate with ASD (Denisova et al., 2013; Denisova et al., 2016). However, some medical information and related important information (i.e., socio‐economic status) were not routinely available and known for all subjects in the NDA, because original studies had different and varying research programmes. Second, data from collections that were recruiting subjects for medical conditions associated with ASD (e.g., Fragile‐X) were not excluded initially to address critiques (e.g., Lee et al., 2021) about the overall lack of generalizability in the ASD field across the entire spectrum of individuals who could be diagnosed with ASD according to the DSM‐5. By not initially excluding subjects with potential medical conditions, this current work aims to characterize cognitive abilities in all individuals with ASD diagnosed in early childhood, without regard to the potential biological cause of the diagnosis. Third, unless the case involves a highly penetrant, loss‐of‐function genetic abnormality (such a full mutation of FMRP in the Fragile‐X Syndrome, FXS), a relationship between a particular genetic abnormality (such as an excess of likely gene disrupting [LGD] de novo mutations or an inherited ancestral variant, or being a premutation carrier of FMRP) and ASD phenotype can only be provided on probabilistic (and not deterministic) terms, and currently cannot be done on an individual level. For instance, some individuals with genetic abnormalities that are associated with ASD diagnoses do not exhibit the expected ASD phenotype and/or do not meet best clinical judgment criteria.

While the initial overall sample subsumed all eligible infants, main analyses were repeated on the set with ascertained outcomes withholding study‐sites recruiting participants with neurogenetic conditions associated with ID and ASD (see below).

The breakdown for the total of 62 dataset identifiers (along with Submitters) are listed in Table S1, and represent contributions from academic institutions or study‐sites (subsumes intramural NIH investigators and investigator networks) from at least 18 US states across the US. With regard to the subject population, the original investigators' recruitment efforts across these 62 study‐sites range from studies involving infants without specific developmental risks and to those at risk or with atypical development, and represent children from families who take part in NIH‐funded research studies in the US.

Specifically, included among the top five study‐sites with the largest number of children assessed on the MSEL (Mullen, 1995), there are studies recruiting infants at risk for atypical development, for example, due to developmental concerns (e.g., pediatrician check‐up around 12 months of age; collection #2115), those at biological or familial (genetic) risk for ASD (e.g., infants at High Risk due to older biological sibling with an ASD; #18), those at genetic and environmental risks for autism (e.g., collection #2066), as well as those infants with no known concerns or any specific risk (e.g., development in twins; #2384). Additional examples of the varied research questions probed by the 62 contributing study‐sites include studies recruiting infants who may have medical conditions known to be associated with ASD (e.g., Fragile‐X Syndrome, FXS; #1888), those with potential environmental exposures to compounds in household products, and for therapeutic intervention studies (Table S1).

All data are de‐identified in compliance with U.S. Health Insurance Portability and Accountability Act (HIPAA) guidelines. Signed written informed parental consent was obtained by original study investigators in accordance with U.S. 45 CFR 46 and Declaration of Helsinki for participation and study procedures were approved by the IRB of each institution. Analyses of these de‐identified data were reviewed and approved by, and a determination of 'not human subjects research' was obtained from, the Institutional Review Board of City University of New York, Queens College. The MSEL (Mullen, 1995) assessments and associated behavioral and clinical outcomes data for each subject are those included in the May 2019 NDA/NDAR data release, with additional clinical outcomes data included in the 2020 and 2021 data releases.

The inclusion criteria for including datasets from specific subjects in the current study consisted of (*i*) availability of at least one assessment on the Mullen (Mullen, 1995) containing raw or Age Equivalent (AE) scores on all four cognitive subscales (required to estimate non‐verbal and verbal subdomain scores; additional details in the section below) and (*ii*) assessments from children (neonates, infants, toddlers, preschoolers) ranging in age from birth up to approximately 68 months of age, an age range for valid administration of the Mullen. The exclusion criteria for assessment data points were duplicate records, implausible scores (e.g., 999, negative values), availability of only standard (T scores) but not raw or AE scores for any of the four cognitive subscales, and failure to reconcile information in a child's record from other sources (e.g., age at assessment). In order to ensure quality of the data entering analyses, rigorous quality checks were performed using both automated scripts and manual procedures, with discrepancies resolved via multiple consultations with NDA/NDAR staff.

The overall sample included 15,030 assessments from *N* = 8065 unique participants, with *N* = 1956 who were assessed on the MSEL longitudinally for at least three or more times (7052 assessments). The sample includes neonates, infants, toddlers, and preschoolers from 2 to 68 months of age (NDAR provides ages in months). For *N* = 8065 unique participants, the Male to Female ratio (M/F) is 5485 M/2580F.

Because this study is focused on examining cognitive abilities vis‐à‐vis ASD manifestations, as a second step, for all *N* = 8065 subjects information was obtained from NDA on all available ADOS assessments and ASD status for each subject. From this overall sample, outcomes were ascertained or ruled out for *N* = 6029 (10,898 assessments) (Table S2). The details on ascertainment are as in the following sections.

### Ascertainment of ASD outcomes

The diagnosis of ASD according to DSM‐5 was made on the basis of the Autism Diagnostic Observation Schedule, ADOS (either the Generic, ADOS‐G; Lord et al., 2000) or ADOS‐2 (Lord et al., 2012, version) administration for all participants, available history and parental interviews, and expert clinical opinion (ADI‐R [Lord et al., 1994] parent interviews were also available for about 85% of subjects who had the ADOS as well as the clinical diagnosis). Revised ADOS algorithms were used (Gotham et al., 2007, 2008). Diagnoses made using the DSM‐IV criteria (Autism, Autism Spectrum Disorder, Pervasive Developmental Delay—Not Otherwise Specified, PDD‐NOS) were converted to DSM‐5 diagnosis of Autism Spectrum Disorder, ASD (American Psychiatric Association, 2013). We computed Calibrated Severity Scores (CSS) (for Social Affect, SA, Restricted and Repetitive Behaviors, RRB, and Total) on the ADOS for all children for whom item‐level scores and language level (A1 item) information were available, according to (Gotham et al., 2009; Hus et al., 2014) (for Modules 1, 2, and 3) and according to (Esler et al., 2015) for the Toddler module (99% of the subjects with ascertained outcomes have CSS scores; Table S3). In case of longitudinal studies with subjects with multiple assessments, the date of the latest diagnostic assessment was used when forming subgroupings. The average age at ADOS assessment for children was around 3 years (on average, about 43 months for ASD, and 30 months for TD [33 months for the third group in which ASD was ruled out]). Note that a few children have an older age at ADOS assessment than this average age, since our inclusion criteria for the Mullen has an upper bound of 68 months (5.6 years).

For risk ratio analyses with *early* Mullen scores obtained when infants were less than 1 year of age, for infants who are subgrouped into ASD and TD groups, the average ages at their earliest available ADOS were similar for the two groups (about 28 months or 2.3 years: 28 months for ASD and 27.5 months for TD).

Of the *N* = 6029 subjects with ascertained outcomes, the focus of the current study is on the two subgroupings: *N* = 3098 participants who are ascertained to have an Autism Spectrum Disorder (ASD) and *N* = 691 who are ascertained to be TD or healthy controls. The remaining *N* = 2240 participants were subsumed into a third group, subsuming participants for whom ASD was ruled out but TD status was not conferred; this group ('noASDdetected') is considered in supplementary analyses. This group includes children with varied developmental or neuropsychiatric concerns of “developmental delay of mixed etiologies” (language delay, motor delay, general delay) and “other” (Attention Deficit Hyperactivity Disorder [ADHD] and learning difficulties), or who were coded as being non‐spectrum (i.e. and who were not also ascertained as being typically developing or healthy controls).

Specifically, ASD was ascertained in *N* = 3098 (5036 assessments), with *N* = 535 children who were assessed longitudinally for at least three or more times (1828 assessments). TD was ascertained in *N* = 691 (1370 assessments), with *N* = 215 who were assessed longitudinally for at least 3 or more times (736 assessments). ASD was ruled out ('noASDdetected') in *N* = 2240 (4492 assessments), with *N* = 638 who were assessed longitudinally for at least 3 or more times (2297 assessments). The male/female proportions for all subgroupings are presented in Table S2.

### Subgroupings vis‐à‐vis neurogenetic conditions associated with intellectual disability

The overall initial sample contained subjects recruited into studies on neurogenetic conditions associated with ID and ASD (e.g., Tuberous Sclerosis, *N* = 158, collection #2008 and Fragile‐X syndrome, *N* = 128, collection #1888). However, the subgroupings of subjects with ascertained outcomes (ASD, TD, noASDdetected) (see above, *N* = 6029), contained no datasets from the Tuberous Sclerosis study‐site. Further, the ASD subgroup contained a small percent (about 2%) of Fragile‐X datasets (from collection #1888) (a small percent of datasets [about 3%] from collection #1888 were also present in the noASDdetected subgroup, but none in the TD group).

To preclude the possibility that the findings reflect the risk for ASD in young children with Fragile‐X syndrome, a neurogenetic condition characterized by low IQ, main analyses were repeated with subgroups from the set without Fragile‐X subjects (the Ns for the two versions [with Fragile‐X and without] are included in the Table S2).

### The Mullen scales of early learning: background and neurobiology

The MSEL (Mullen, 1995) assesses abilities on five domains: Receptive Language (RL), Expressive Language (EL), Visual Reception (VR), Fine Motor (FM), and Gross Motor (GM) scales (GM is given up to 33 months). The focus of the current study is on the RL, the EL, the VR, and the FM scales which represent four cognitive scales of MSEL (Mullen, 1995), and the Early Learning Composite (ELC) standard score, a “summative measure of g” (Mullen, 1995) (constructed using the four cognitive scales; see below for detailed description).

The MSEL battery is guided by the information processing approach to human mind, brain, and behavior, and is rooted in the importance of motor development for overall child development (Mullen, 1995). That the MSEL is developed around the motor base and motor milestones is significant, and supported by foundational theoretical and empirical studies on the importance of primacy of sensorimotor development for children's sensorimotor, perceptual, and cognitive competence. Of note, we (Denisova, 2019; Denisova & Zhao, 2017) and others (Iverson et al., 2019) have detected neurobiological evidence supporting the framework of the MSEL to study emergence of autism. For instance, in a longitudinal study, lower performance on RL between 6 and 36 months is linked to atypical social development of the child (ASD manifestations at 36 months), and to atypical sensorimotor functioning at 9 months (Denisova, 2019).

The view that different cognitive abilities (e.g. nonverbal vs verbal) may be subserved by the relatively distinct neural processes in the brain permits a relative separation of distinct abilities to be measured with different subscales, and further permits direct comparison and investigation of subdomain estimates, one of the goals of the current study. This ‘modular’ approach is reflected in MSEL's structure that enables organization of information subsumed by these two subdomains, nonverbal vs. verbal (Mullen, 1995) (According to Wasserman & Tulsky, 2005, Wechsler is credited with separating verbal and performance into distinct scales). We therefore look at the separate domains subsuming nonverbal and verbal abilities on MSEL.

MSEL can be given as early as a few days after birth (0–68 months), in contrast to other traditional measuring scales of cognitive abilities (differential abilities scales, DAS‐II; Elliot, 2007; Stanford‐Binet‐SB5; Roid, 2003) which are given around the 2nd year of life. Because MSEL can be used very early life, this measure can provide useful, potentially prognostic, information about whether a certain subset of children (i.e., those who go on to develop ASD) have atypically variable or uneven cognitive abilities profile as a function of age. This is an important goal, because although reliable and established ASD diagnostic assessments can be given in the 2nd and 3rd year of life, we currently lack neurobiologically grounded, valid and reliable tools for detecting ASD in the 1st year of life—the time when opportunities for supports and intervention would be most helpful. Thus, improved understanding of features of cognitive profiles early in life may serve the goal of developing understanding and new insights into early ASD precursors. Specifically, uneven progress in learning and skill acquisition can be detected using subdomains on the MSEL starting soon after birth and tracked using this single instrument to preschool age (an important advantage, since switching instruments may introduce false changes in ability estimates; cf. Farmer et al., 2016). More fundamentally, we can characterize the level of cognitive abilities in children receiving ASD diagnoses in early childhood.

### An “age gradations” approach for item construction for MSEL

A significant feature of MSEL's construction and administration is that test items are arranged and presented in the order of increasing difficulty, with the test items developed, classified and grouped as a function of nine age gradations (after Stanford‐Binet Intelligence Scale 1960 Version L‐M edition; Terman & Merrill, 1960; cf. Mullen, 1995, p. 5) and consistent with the 1908 Binet‐Simon version of the graded method tests (in Binet & Simon, 1916, p. 237). The graded method tests make it possible to estimate the child's intellectual level or mental age, which indicates that “the intelligence of the child tested is equivalent to the average intelligence of the children of the age stated” (Stern, 1914, p. 38). However, numerically equivalent values (or differences in values), at different ages, are not psychologically identical. This is a fundamental challenge when seeking to make meaningful comparisons of a given numerical value (e.g. mental age) at different chronological (e.g., physical) ages.

Historically, to address this challenge, Stern introduced the concept of a mental quotient (MQ), which involves dividing the mental age by the chronological age (Stern, 1914, pp. 42 and 80). Expressing the mental quotient, MQ as a ratio makes the MQ value somewhat independent of chronological age (Stern, 1914) (MQ is referred to as ratio intelligence quotient [IQs] by subsequent researchers). Note that in later approaches to cognitive abilities testing, the field shifted from computing ratio IQs (or MQs) to deviation IQs (“standardized normative mean of 100 and SD of 16” (Roid & Barram, 2004), p. 7, introduced by Simon‐Binet IS, 3rd Revision (Terman & Merrill, 1960)), although this computation and normalization *per se* is not part of the MSEL. Importantly, however, IQ estimates computed as ratio IQs on the Mullen are highly correlated with estimates on the DAS (e.g., Bishop et al., 2011) and DAS‐II (Farmer et al., 2016), which we confirm for a subset of subjects in this data set with available scores on both DAS‐II and MSEL assessments (see below). The Age Equivalent (AE) scores on the MSEL generally parallel the concept of intellectual level or mental age pioneered by seminal work of Simon & Binet (Binet & Simon, 1916). AEs indicate the “age at which the child's raw score is the median score” (Mullen, 1995, p. 35). The division by the child's chronological age in the formulas below parallels the MQ concept introduced by Stern (Stern, 1914) and yields a ratio IQ.

### Estimates of intelligence quotient on MSEL: verbal and non‐verbal abilities, and a summative measure of *g*

The estimates of a verbal developmental quotient (vDQ) (henceforth, verbal intelligence quotient, VIQ) require AE scores, from the RL and EL verbal domains, while the estimates of a non‐verbal developmental quotient (nvDQ; henceforth, performance intelligence quotient, PIQ) involve the VR and FM non‐verbal domains. For each of the four scales, we computed AEs using original raw scores using conversion algorithms (Table C4 in the manual, Mullen, 1995), for participants with raw scores. Available AE scores were used directly only when raw scores were not provided by the original investigators.

The verbal intelligence quotient (VIQ) domain estimate for each child is calculated by adding and averaging Receptive Language (RL) and Expressive Language (EL) AE scores, dividing by chronological age, and multiplying by 100:

(1) $$\mathrm{VIQ}=\left(\left(\left({\mathrm{RL}}_{\mathrm{AE}}+{\mathrm{EL}}_{\mathrm{AE}}\right)/2\right)/\text{chronological age}\right)\times 100.$$

The nonverbal or performance intelligence quotient (PIQ) domain estimate for each child is calculated by adding and averaging Visual Reception (VR) and Fine Motor (FM) AE scores, dividing by chronological age, and multiplying by 100:

(2) $$\mathrm{PIQ}=\left(\left(\left({\mathrm{VR}}_{\mathrm{AE}}+{\mathrm{FM}}_{\mathrm{AE}}\right)/2\right)/\text{chronological age}\right)\times 100.$$

This process of constructing verbal and non‐verbal (“performance”) estimates from MSEL AEs has been used in a number of developmental studies, in particular, in studies examining the IQ profile on the Mullen (e.g., Rogers et al., 2003; Short et al., 2013) (note that subdomain T‐scores are available on the MSEL but are not used in this study due to floor effects). An overall developmental quotient (DQ), taking into account AEs from four cognitive scales, is calculated as:

(3) $$\mathrm{DQ}=\begin{array}{l} \left(\left(\left({\mathrm{RL}}_{\mathrm{AE}}+{\mathrm{EL}}_{\mathrm{AE}}+{\mathrm{VR}}_{\mathrm{AE}}+{\mathrm{FM}}_{\mathrm{AE}}\right)/4\right)/\text{chronological age}\right)\\ \times 100. \end{array}$$

An Early Learning Composite (ELC, a standard score), a “summative measure of g” (Mullen, 1995), is considered for all participants for whom it could be computed. The ELC is a standard score, derived using Cognitive T score sum of the four “cognitive” scales of RL, EL, VR, and FM (mean 100, SD: 15).

### Concurrent validation analyses of estimates on MSEL and DAS‐II

In the current study, we examined concurrent validity of cognitive abilities estimates on the MSEL with the DAS‐II (Elliot, 2007) in children assessed on both instruments. The Early Years Battery (EYB) is administered to children between 2:6 and 8:11 years and has two subcomponent levels, the Lower Level (LL; 2:6–3:5 years) and the Upper Level (UL; 3:6–8:11 years); both yield a Verbal Domain standard score and a Nonverbal Domain standard score. In addition, DAS‐II‐EYB provides an overall estimate of complex conceptual abilities via the cognitive ability score (GCA) standard score. The GCA is comparable to the ELC on the MSEL. Estimates of cognitive abilities obtained using DAS‐II ((Elliot, 2007); Technical Manual) have been validated against other widely used measures with children, including Wechsler Preschool & Primary Scale of Intelligence—Third Edition (WPPSI‐III) (Wechsler, 2002) and Wechsler Intelligence Scale for Children—Fourth Edition (WISC‐IV) (Wechsler, 2003). A total of *N* = 90 children were assessed on DAS‐II–EYB up to 68 months of age, with both verbal and non‐verbal standard scores and the GCA standard scores. About 96% of children were assessed on the same day with both the MSEL and DAS‐II (4 out of 90 were assessed within the 30 days). We performed Pearson correlations (two‐tailed) between non‐verbal standard score (on DAS‐II) and PIQ estimates (on the MSEL), as well as correlations between verbal standard score (on DAS‐II) and VIQ estimates (on the MSEL). Correlations were also performed between the standard scores reflecting general cognitive abilities of the child: the GCA from the DAS‐II and the ELC from the MSEL.

### Statistical analyses

The functions and tools in the Statistics and Machine Learning and Curve Fitting Toolboxes in MATLAB (MathWorks) were used for all statistical analyses. MATLAB's fitlme function was used to fit a linear mixed‐effects (multilevel) model, with the maximum likelihood (ML) estimation method for parameter estimation. Age was the predictor and IQ was the response variable, and each child's data modeled separately. Specifically, the multilevel model included varying intercepts and slopes for each child, in the form: *y* ~ 1 + *X*1 + (1 + *X*1|*g*1) (same as: *y* ~ *X*1 + (*X*1|*g*1)). This model accounts for potential differences in time trends for children with more than one time point of IQ assessment. Separate models were run for the different diagnostic groups (overall sample, and ASD, TD, and noASDdetected) and for distinct IQ estimates (ELC, and AE‐based estimates of IQ: PIQ, VIQ, and DQ). The linear fits for IQ scores vs. age (fixed effect) are plotted with a 95% confidence band; the estimated coefficients from the multilevel analyses (slope and intercept) are given with 95% confidence intervals (CIs). The focus of the current work is on ASD and TD outcomes; supplementary analyses are conducted for subjects for whom ASD is ruled out ('noASDdetected'). A Wilcoxon rank sum test (two‐tailed) tested whether the IQ medians are different between ASD and TD subgroups (alpha of 0.05 was used for all tests).

The age bins analysis was done by grouping ASD and TD cases by age, at 6, 12, 18, 24, 30, and 36 months (±1 month), separately for longitudinal subjects who had 3 or more time points on the Mullen, and for a separate set of ‘singletons’ with only a single time point on the Mullen. The longitudinal set consisted of the following Ns at each age bin: 6 months (*N*_ASD_ = 158, *N*_TD_ = 54), 12 months (*N*_ASD_ = 212, *N*_TD_ = 119), 18 months (*N*_ASD_ = 117, *N*_TD_ = 45), 24 months (*N*_ASD_ = 159, *N*_TD_ = 98), 30 months (*N*_ASD_ = 69, *N*_TD_ = 41), 36 months (*N*_ASD_ = 160, *N*_TD_ = 69). For the singleton age bin set, the Ns were: 6 months (*N*_ASD_ = 6, no TD cases), 12 months (*N*_ASD_ = 20, *N*_TD_ = 42), 18 months (*N*_ASD_ = 87, *N*_TD_ = 50), 24 months (*N*_ASD_ = 93, *N*_TD_ = 25), 30 months (*N*_ASD_ = 53, *N*_TD_ = 18), 36 months (*N*_ASD_ = 48, *N*_TD_ = 3).

Several analyses explored further the association between cognitive abilities on the full‐scale IQ on the ELC standard score (as well as the AE‐based estimates DQ, both from the first assessment of the Mullen) relative to the autism severity on the ADOS using the calibrated severity scores (CSS) (social, non‐social, and total CSS) (Pearson's correlation, two‐tailed).

## RESULTS

### Concurrent validity of cognitive ability estimates on MSEL and DAS‐II (EYB)

We first wanted to demonstrate the extent to which cognitive abilities estimates from the Mullen Scales of Early Learning (MSEL, Mullen, 1995) correlate with estimates from another widely used assessment of cognitive abilities in children, the Differential Abilities Scale (DAS‐II, Elliot, 2007). In this study, strong concurrent validity is observed for cognitive abilities estimated on the MSEL and DAS‐II (Elliot, 2007) for 90 children from the overall sample who were assessed on both instruments. The general ability estimates (cognitive ability score, GCA on DAS‐II vs. ELC on the Mullen) are significantly correlated, *r*(88) = 0.8848, *p* = 6.2993e−31. The domain scores on the two assessments are also significantly correlated, both for non‐verbal (*r*(88) = 0.7384, *p* = 9.9220e−17) and for verbal estimates (*r*(88) = 0.8424, *p* = 2.3751e−25).

### Reliability analyses: early versus later time points

We calculated the reliability of early test scores for children, again from the overall sample, who were tested at 6 months and at later time points. This was done in order to see how trustworthy any conclusions might be, particularly regarding early IQ estimates vis‐à‐vis future ASD diagnosis. That is, are early low or high scores just as reliable over time as average scores? The reliability computations between 6 and the 12, 18, 24, 36 months (VIQ, PIQ, and ELC) indicate correlations around *r* = 0.30 (overall sample, *p* < 0.01) (Figure S1). Further, the correlations between early low IQ scores (i.e., those below the median) remained significant (*p* < 0.01), indicating that early low IQ estimates are especially stable over time. Thus, this stability of the scores over time provides the basis for relating early IQ data to later ASD ascertainment.

Note that the reliability analyses combined data from males and females with ASD, based on preliminary analyses at 6 months of age that examined mean cognitive abilities estimates of males and females separately. Specifically, while the ELC score at 6 months was significantly lower than the population mean of 100 when both males and females are combined, no differences were detected between males and females with ASD (Figure S2; consistent pattern for AE‐based DQ estimate).

### Preliminaries: overall sample

Because this is an unselected sample from the NDA, with children receiving assessments on cognitive abilities continuously at different time points between the ages of 2 and 68 months, the IQ data are initially examined as a function of age, and then as a function of outcome status. This was done to ensure that age effects, if any, are fully accounted for. In particular, the full‐scale IQ standard scores (ELC) are expected to stay relatively stable over time, because these scores are normed to have a mean of 100 and SD of 15. The Age Equivalent (AE) scores are not expected to worsen with time (and may show improvement). However, a worsening or some deviation from the typical or normative pattern over time may be notable, potentially signaling the presence of children with low cognitive abilities. This question has not been probed previously over such a wide age range for a single instrument (MSEL, Mullen, 1995). Moreover, in subsequent analyses below (in the next section), pre‐specified age bins are created to compare cases grouped by age and by outcome.

As expected, an overall flat pattern over time is observed for the standard, normed ELC IQ scores in the overall sample (95% CIs overlap 0: *B*_slope_ = −0.01 [−0.03 0.02], *p* = 0.68). That the ELC does not fluctuate with age when all children are considered (regardless of diagnostic outcome) indicates the relative stability of the assessment across the different ages, consistent with expectation. Note that the intercept is about 92 (in contrast to the population mean of 100); this lower value may be due to the presence of lower‐IQ data points at all ages for a relatively large subset of children (Table S4 presents additional results for the overall sample, using DQ [AE‐based full‐scale IQ], as well as AE‐based subdomain scores of VIQ and PIQ**).** This pattern reflects the likely enrichment of this sample with likely (or at‐risk) cases of ASD or other disorders of neurodevelopment.

### For children with ascertained outcomes: focus on ASD and TD group differences in IQ trajectories with age

**FIGURE 1:**
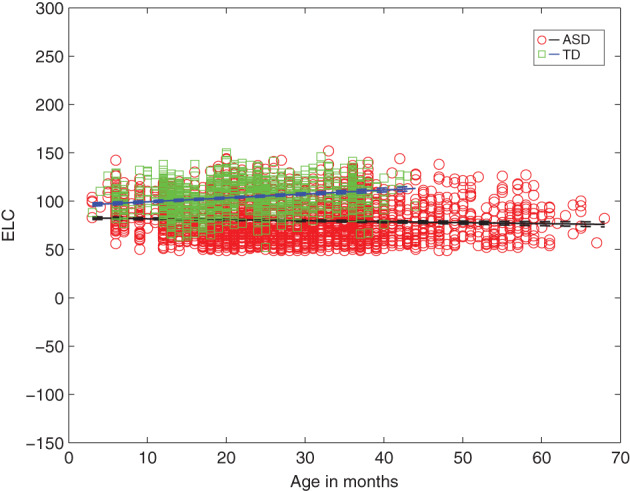
Intelligence quotient (IQ) (ELC) as a function of age for infants from ASD versus TD groups from linear multilevel models, showing significantly lower *y*‐intercept and a negative slope for ASD (vs. TD) as a function of age. ELC, Early Learning Composite standard score (mean 100, SD: 15); ASD, Autism Spectrum Disorder; TD, typically developing. In children with ASD, increased age predicts significantly lower full‐scale IQ (ELC) (*B*_slope_ = −0.10, *t*[2943] = −3.53) while in TD children, increased age predicts significantly higher ELC (*B*_slope_ = 0.40, *t*[1179] = 8.91) (all *p* < 0.001)

For children with ascertained outcomes, Figure 1 shows that as a group, those with ASD have a significantly *lower* intercept for full‐scale IQ (*g*, ELC standard score) as well as a *shallower* negative slope with increasing age, relative to TD children. Specifically, non‐overlapping 95% CIs are detected for parameter estimates for both the intercept and slope in ASD versus TD for ELC (*B*_interceptASD_ = 82.74 (81.18 84.30) vs. *B*_interceptTD_ = 95.22 (93.26 97.18); *B*_slopeASD_ = −0.10 (−0.15 0.04) vs. *B*_slopeTD_ = 0.40 (0.31 0.49)). Age explains about 66% of variance in ELC in ASD (vs. 41% in TD) (Table 1). (Consistent results are detected for DQ estimates [AE‐based full‐scale IQ] for ASD vs. TD group; Figure S3 and Table S5).

**TABLE 1:** Parameter estimates from the linear multilevel analyses: intelligence quotient (IQ) (Early Learning Composite [ELC] standard score) and age for Autism Spectrum Disorder (ASD) and typically developing (TD) subgroups

ASD IQ (ELC)							
Fixed effects coefficients (age)	Parameter Estimate	SE	95% CIs		df	*t* value	*p* value
*β*_0_ intercept (grand‐average)	82.74	0.79	81.18	84.30	2943	103.89	<0.001
*β*_1_ slope (grand‐average)	−0.10	0.02	−0.15	−0.04	2943	−3.53	<0.001
Random effects covariance (child)	Parameter Estimate	95% CIs					
Intercept (SD)	13.49	11.38	15.98				
Age (SD)	0.43	0.35	0.53				
Correlation: age, intercept	−0.41	−0.58	−0.20				
Observations	2945						
*R* ^2^	0.66						
Adj. *R* ^2^	0.66						
*F* statistic	12.843 (df = 1, 2943), *p* = 0.00034428						
Resid. SD	11.53 (10.97 12.2 95% CIs)						

TD IQ (ELC)							
Fixed effects coefficients (age)	Parameter Estimate	SE	95% CIs		df	*t* value	*p* value
*β*_0_ intercept (grand‐average)	95.22	0.99	93.26	97.18	1179	95.25	<0.001
*β*_1_ slope (grand‐average)	0.40	0.04	0.31	0.49	1179	8.91	1.7447e−18
Random effects covariance (child)	Parameter Estimate	95% CIs					
Intercept (SD)	7.71	4.73	12.57				
Age (SD)	0.31	0.20	0.49				
Correlation: age, intercept	−0.20	−0.70	0.43				
Observations	1181						
*R* ^2^	0.41						
Adj. *R* ^2^	0.41						
*F* statistic	79.562 (df = 1, 1179), *p* = 1.7447e−18						
Resid. SD	10.92 (10.19 11.71 95% CIs)						

*Note*: The ELC is the standard composite score on the Mullen, providing an estimate of IQ or *g*.

**FIGURE 2:**
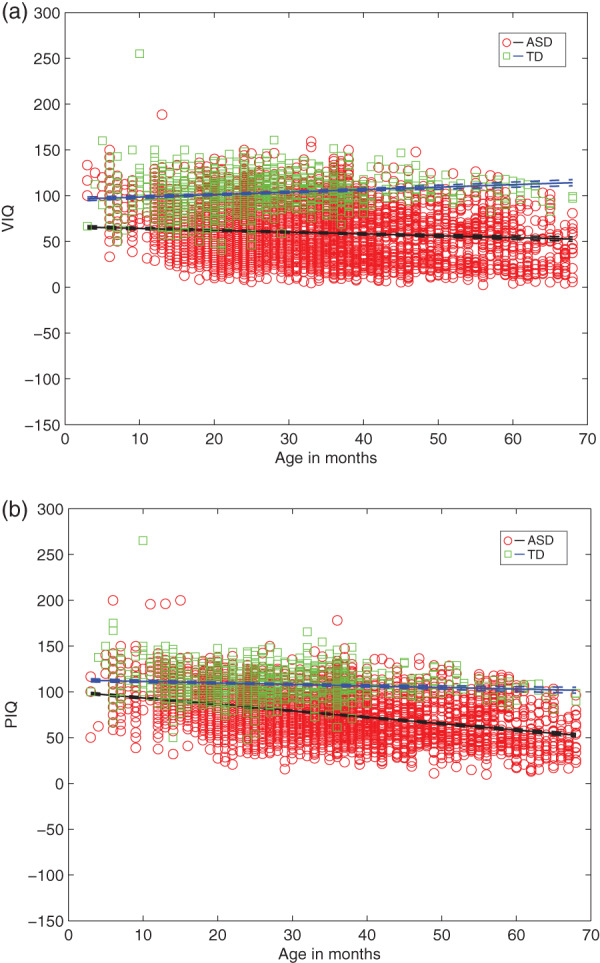
The VIQ and PIQ estimates as a function of age for infants from ASD versus TD groups from linear multilevel models, showing lower y‐intercept and a negative slope for ASD (vs. TD) with increasing age. (a) VIQ for ASD, TD and (b) PIQ for ASD, TD. VIQ, verbal intelligence quotient; PIQ, performance intelligence quotient; ASD, Autism Spectrum Disorder; TD, typically developing. In children with ASD, increased age predicts significantly lower VIQ (*B*_slope_ = −0.19, *t*[5034] = −7.08) and lower PIQ (*B*_slope_ = −0.69, *t*[5034] = −30.34) (all *p* < 0.001). In TD children, increased age predicts significantly higher VIQ (*B*_slope_ = 0.27, *t*[1368] = 6.96) and lower PIQ (*B*_slope_ = −0.16, *t*[1368] = −4.653) (all *p* < 0.001)

These between group differences also hold for AE‐based domain scores of VIQ and PIQ (Figure 2 **)**, with ASD showing lower VIQ as well as PIQ relative to TD. The parameter estimates for both the intercept and slope are significantly different (non‐overlapping 95% CIs) for ASD versus TD, for VIQ: (*B*_interceptASD_ = 66.20 (64.39 68.01) vs. *B*_interceptTD_ = 95.77 (93.55 97.99); *B*_slopeASD_ = −0.19 (−0.25 0.14) vs. *B*_slopeTD_ = 0.27 (0.19 0.34)), and for PIQ: (*B*_interceptASD_ = 100.24 (98.82 101.66) vs. *B*_interceptTD_ = 113.04 (111.12 114.96); *B*_slopeASD_ = −0.69 (−0.74 0.65) vs. *B*_slopeTD_ = −0.16 (−0.23 0.09)). In ASD age explains about 75% in VIQ (vs. 31% in TD) and 81% of variance in PIQ (vs. 21% in TD) (Table 2).

**TABLE 2:** Parameter estimates from the linear multilevel analyses: age and verbal intelligence quotient (VIQ), and age and performance IQ (PIQ) for Autism Spectrum Disorder (ASD) and typically developing (TD) subgroups

ASD							
VIQ							
Fixed effects coefficients (age)	Parameter Estimate	SE	95% CIs		df	*t* value	*p* value
*β*_0_ intercept (grand‐average)	66.20	0.92	64.39	68.01	5034	71.82	<0.001
*β*_1_ slope (grand‐average)	‐0.19	0.02	−0.25	‐0.14	5034	−7.05	1.9912e−12
Random effects covariance (child)	Parameter Estimate	95% CIs					
Intercept (SD)	21.63	19.33	24.20				
Age (SD)	0.57	0.48	0.67				
Correlation: age, intercept	‐0.43	‐0.56	‐0.28				
Observations	5036						
*R* ^2^	0.75						
Adj. *R* ^2^	0.75						
*F* statistic	49.74 (df = 1, 5034), *p* = 1.9912e−12						
Resid. SD	15.73 (15.05 16.45 95% CIs)						
PIQ							
Fixed effects coefficients (age)	Parameter Estimate	SE	95% CIs		df	*t* value	*p* value
*β*_0_ intercept (grand‐average)	100.24	0.72	98.82	101.66	5034	138.81	<0.001
*β*_1_ slope (grand‐average)	‐0.69	0.02	‐0.74	‐0.65	5034	‐30.34	7.331e‐186
Random effects covariance (child)	Parameter Estimate	95% CIs					
Intercept (SD)	17.66	16.04	19.45				
Age (SD)	0.56	0.50	0.62				
Correlation: age, intercept	−0.62	−0.69	−0.54				
Observations	5036						
*R* ^2^	0.81						
Adj. *R* ^2^	0.81						
*F* statistic	920.61 (df = 1, 5034), *p* = 7.331e−186						
Resid. SD	12.05 (11.59 12.53 95% CIs)						

TD							
VIQ							
Fixed effects coefficients (age)	Parameter Estimate	SE	95% CIs		df	*t* value	*p* value
*β*_0_ intercept (grand‐average)	95.77	1.13	93.55	97.99	1368	84.65	<0.001
*β*_1_ slope (grand‐average)	0.27	0.03	0.19	0.34	1368	6.96	4.9192e−12
Random effects covariance (child)	Parameter Estimate	95% CIs					
Intercept (SD)	15.39	12.60	18.80				
Age (SD)	0.29	0.17	0.48				
Correlation: age, intercept	−0.90	−0.96	−0.70				
Observations	1370						
*R* ^2^	0.31						
Adj. *R* ^2^	0.31						
*F* statistic	48.57 (df = 1, 1368), *p* = 4.9192e−12						
Resid. SD	13.34 (12.56 14.16 95% CIs)						
PIQ							
Fixed effects coefficients (age)	Parameter Estimate	SE	95% CIs		df	*t* value	*p* value
*β*_0_ intercept (grand‐average)	113.04	0.97	111.12	114.96	1368	115.52	<0.001
*β*_1_ slope (grand‐average)	−0.16	0.03	−0.23	−0.09	1368	−4.653	3.5882e−06
Random effects covariance (child)	Parameter Estimate	95% CIs					
Intercept (SD)	10.45	7.86	13.89				
Age (SD)	0.20	0.06	0.59				
Correlation: age, intercept	−0.74	−0.90	−0.43				
Observations	1370						
*R* ^2^	0.21						
Adj. *R* ^2^	0.21						
*F* statistic	21.65 (df = 1, 1368), *p* = 3.5882e−06						
Resid. SD	12.52 (11.78 13.31 95% CIs)						

In addition, consistent findings are detected when the ASD group is compared to the group in which ASD (and not another developmental disorder) was ruled out but for which TD status was not established (noASDdetected). Specifically, those with ASD have a significantly lower intercept for full‐scale IQ (g, ELC standard score) as well as a shallower negative slope with increasing age, relative to this noASDdetected group (Figure S3C). Of note, noASDdetected group also has a significantly lower intercept as well as a shallower negative slope with increasing age on the ELC relative to TD group (Table S6 presents parameter estimates from multilevel models of age with IQ for noASDdetected group).

### Pattern of low IQ in ASD persists when datasets from neurogenetic conditions are withheld from analysis

The analysis excluding a small number of datasets from study‐sites focusing on neurogenetic conditions associated with ID and ASD yielded similar parameter estimates for all three groups with ascertained outcomes. Specifically, Table S7 (noASDdetected subgroup without Fragile‐X) and Table S8 (ASD subgroup without Fragile‐X) present parameter estimates results, which were unchanged, when Fragile‐X datasets were excluded from the sample; note that TSC datasets were not included in the sets with ascertained outcomes. (TD subgroup contained no subsets from either TSC or Fragile‐X). *This analysis shows that participants with the lowest IQ scores do not come from studies that recruited families with neurogenetic conditions*.

### Analysis by age bins: 6, 12, 18, 24, 30, 36 months focusing on *g* (ELC) for ASD and TD at selected ages

**FIGURE 3:**
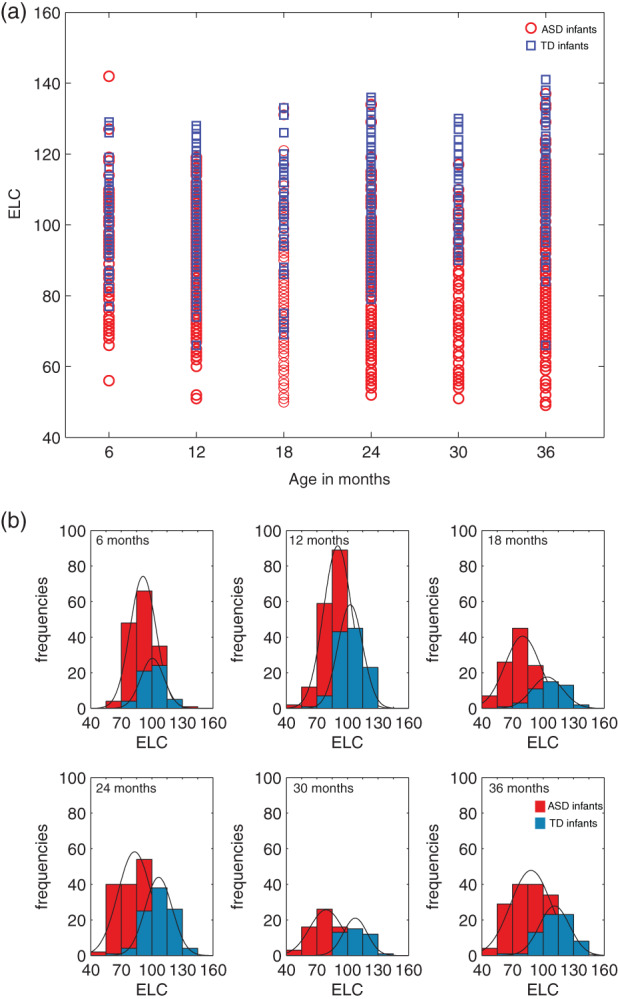
Intelligence quotient (IQ) (ELC) by age groupings: age bins and frequency histograms for infants with ASD and TD outcomes. Data from infants with at least three or more assessments on the Mullen, such that at least two of the assessments fall within the pre‐defined age bins. (a) Age bins (6, 12, 18, 24, 30, and 36 ± 1 months). (b) For each of the age bins in (a), frequency and probability density function (PDFs) are shown. ASD, Autism Spectrum Disorder; TD, typically developing. The width of the bars is 15, equal to the SD of the Early Learning Composite (ELC) standard score (mean 100)

We next asked if IQ scores are consistently lower in ASD relative to TD when cases are grouped by selected ages. Figure 3 shows the frequency histograms and PDFs for ELC at each of the age bins, from a subset of subjects with three or more assessments, with at least two data points from the same infant falling into the bins. We detected significant differences in ELC scores in ASD versus TD, such that IQ is significantly lower (*p* < 0.001) in ASD relative to TD at each of the age bins considered.

Specifically, a Wilcoxon rank sum test indicated that at 6 months, the median ELC ranks for *ASD*, Mdn = 92, are significantly lower than the median TD ranks, Mdn = 101, *Z* = −4.6564, *p* = 3.2174e−06. At 12 months, the median ranks for ASD were significantly lower than TD (ASD, Mdn = 91; TD, Mdn = 102), *Z* = −4.6564, *p* = 6.7273e−14. Between group differences were significant also at 18 months (ASD, Mdn = 79; TD, Mdn = 107, Z = −6.93072, *p* = 4.1881e−12), at 24 months (ASD, Mdn = 83; TD, Mdn = 107, Z = −9.8807, *p* = 5.0491e−23), at 30 months (ASD, Mdn = 78; TD, Mdn = 107, *Z* = −7.3713, *p* = 1.6900e−13), and at 36 months (ASD, Mdn = 88; TD, Mdn = 112, *Z* = −7.5742, *p* = 3.6122e−14).

For the sake of robustness, to see if the findings are consistent for ‘singletons’ (infants with 1 and only 1 Mullen assessment), age bin analyses were conducted on this separate set, at each of the bins considered for which data from both ASD and TD were available (12, 18, 24, 30, and 36 months: Figure S4). The pattern of results from singletons analysis (this set had fewer subjects available per bin, but had at least *N* = 18 per group, per bin at 12, 18, 24, and 30 months), is consistent with the findings on the repeatedly tested children. The median ranks for ASD were significantly lower than TD at 12 months (ASD, Mdn = 82.5; TD, Mdn = 98.5, *Z* = −4.3704, *p* = 1.2401e−05), at 18 months (ASD, Mdn = 72; TD, Mdn = 95, *Z* = −6.6190, *p* = 3.6152e−11), at 24 months (ASD, Mdn = 73; TD, Mdn = 111, *Z* = −5.6550, *p* = 1.5585e−08), at 30 months (ASD, Mdn = 70; TD, Mdn = 103.5, *Z* = −5.0639, *p* = 4.1071e−07), and at 36 months (ASD, Mdn = 69.5; TD, Mdn = 108, *Z* = −2.4638, *p* = 0.0137).

### Risk ratio for ASD is higher in infants with low IQ, and in particular, early low IQ

Thus far, we detected evidence for relative stability over time of early low IQ scores and significantly lower IQ scores for infants in the ASD group relative to TD group (at each age bin considered, including at 6, 12, 18, 24, 30, and 36 months). These findings suggest that a lower IQ (and not necessarily a lower VIQ score), in particular at an early age, can be considered a potential marker of future ASD diagnosis. In the next analysis, we sought to express this observation in a more formal manner, using a risk ratio (RR). Are the measures obtained on the Mullen predictive of ASD?

In this study, infants who scored below 2 SD on the IQ (ELC, here and below, scores at first assessment are used) had 1.4 times the risk of ASD diagnosis compared to infants who did not score below 2 SD (Risk Ratio 1.48 [1.40 1.56 95% CIs]). This higher risk ratio holds when males and females were analyzed separately.

#### Extremely early IQ

In a follow‐up analysis including infants less than about 1 year of age, those who scored below 2 SD on the ELC had 1.4 times the risk of ASD diagnosis compared to infants who did not score below 2 SD (risk ratio 1.42 [1.20 1.69 95% CIs]). This higher risk ratio is significant in male infants, but not in female infants. Subsequent analysis using AE‐based scores focused specifically on this early life period, across all male and female infants combined. Again, higher risk ratios were observed when using AE‐based IQ estimates, in particular, the Developmental Quotient, DQ, and subdomain estimates VIQ and PIQ. Infants less than about 1 year of age who scored below 2 SD on DQ (as well as VIQ and PIQ), had increased risk of ASD diagnosis, consistent for DQ (risk ratio 1.45 [1.18 1.77 95% CIs]), VIQ (risk ratio 1.47 [1.25 1.73 95% CIs]) and PIQ (risk ratio 1.51 [1.36 1.69 95% CIs]).

In summary, we found significantly higher RR for ASD diagnosis due to a lower IQ score at an early age, whether using the standard full‐scale ELC (g) or AE‐based DQ, as well as AE‐based VIQ and PIQ. A young child with a low IQ taken at random from the study sample is 40% more likely to be later diagnosed with ASD than children in this study sample who have average or above IQ.

##### Is full‐scale IQ a more robust and reliable measure?

The findings reported so far suggest that the information provided by full‐scale IQ is not inferior to domain‐based scores. In particular, the VIQ may be less important than previously suspected, indicating that ‘language first’ hypothesis is weakened, at least for the early period and in this sample. We do not know the cause of the low cognitive abilities, which could be due to genetic or other defects.

There is nothing in these data to make a special case of one of the subset of the scales being somehow more predictive forward in time. These data indicate that there is no case to be made for VIQ being more predictive of childhood ASD. It may be that previously, investigators have noticed poorer language‐related skills because they might have expected poorer language (i.e., poorer language skills associate with autism), and might not have noticed poor performance on other tasks. These early cognitive abilities data, that precede ASD diagnoses, show poor early performance on non‐language tasks, not only on verbal tasks. Full‐scale IQ is more reliable because it contains more information about child’ behavior on both verbal and non‐verbal probes. The data above show that the full‐scale IQ is not inferior to predict childhood ASD cases. VIQ is not more predictive of early ASD cases.

With regard to examining age‐related trajectories of cognitive abilities of early‐diagnosed ASD and TD children presented in the earlier section, the slope is as informative as the intercept in each function. Both the slope and intercept must be taken into account at the same time when investigating the nature of cognitive abilities. For instance, focusing only on the relatively large differences in VIQ intercepts between ASD versus TD groups may obfuscate the extremely rapid concomitant decline in PIQ in the ASD group relative to TD group. Specifically, note the extremely steep decline in scores (−0.69) on PIQ in the ASD group (relative to −0.19 for VIQ), which can be contrasted to the relatively low intercept on the VIQ (AE score of 66) (relative to PIQ of 100). This rapid decline in PIQ in the ASD group, represented by the slope, cannot be ignored. In contrast, considering the data from the children's full‐scale IQ, which incorporates both verbal‐ and non‐verbal items and is a standard score, the ELC intercept is significantly lower, and the slope declines more rapidly, in ASD relative to TD group.

### IQ negatively and significantly associates with core autism features

We next probed whether there are specific associations between IQ and core autism features in children with ASD. This analysis was done with ASD cases with at least two Mullen assessments (using scores at first instance) and repeated in a separate set of children, the singleton cases (with 1 and only 1 Mullen assessment). Based on the results reported above, the focus is on the full‐scale IQ (ELC standard scores), with supporting analyses with DQ (AE‐based scores).

**FIGURE 4:**
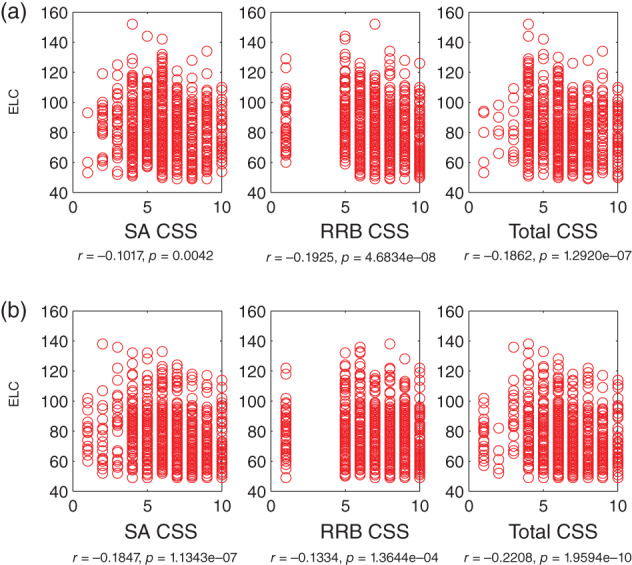
Lower intelligence quotient (IQ) significantly associates with worse autism symptoms: Early Learning Composite (ELC, a standard score) versus Social Affect (SA), Repetitive and Restricted Behaviors (RRB) and Total Calibrated Severity Scores (CSS) on the ADOS (all *p* < 0.01). (a) Data from infants (*N* = 793) with ASD outcomes, with at least two Mullen assessments (the Mullen scores at the 1st time point are chosen for correlation analyses), (b) infants (*N* = 813) with ASD outcomes, with 1 and only one Mullen assessment (no overlap with subjects relative to those in (a)). ASD, Autism Spectrum Disorder; ADOS, Autism Diagnostic Observation Schedule

Lower full‐scale IQ (ELC) scores significantly associate with *worse* social, non‐social, and total Calibrated Severity Scores (CSS) (on the Social Affect, SA, Repetitive and Restrictive Behaviors, RRB, and Total CSS on the ADOS: Figure 4). Specifically, this negative association is robust in ASD cases with at least two Mullen assessments (Figure 4a **)** (ELC and SA CSS, *r*(791) = −0.10 (−0.17 0.03), ELC and RRB CSS *r*(791) = −0.19 (−0.25 0.12), and ELC and Total CSS: *r*(791) = −0.18 (−0.25 0.11), all *p* < 0.01). Moreover, a consistent pattern is observed as well as in a separate set of subjects, in singleton cases with 1 and only 1 Mullen assessment (Figure 4b) (ELC and SA CSS, *r*(811) = −0.18 (−0.25 0.11), ELC and RRB CSS *r*(811) = −0.13 (−0.20 0.06), and ELC and Total CSS: *r*(811) = −0.22 (−0.28 0.15), all *p* < 0.001).

In addition, this pattern of significant negative associations holds when using the DQ, AE‐based full‐IQ estimate, in the longitudinal (DQ and SA CSS, *r*(791) = −0.18 (−0.24 0.12), DQ and RRB CSS *r*(791) = −0.26 (−0.32 0.21), and DQ and Total CSS: *r*(791) = −0.26 (−0.32 0.21), all *p* < 0.01) (Figure S5a) and singleton sets (DQ and SA CSS, *r*(811) = −0.27 (−0.31 0.23), DQ and RRB CSS *r*(811) = −0.18 (−0.22 0.14), and DQ and Total CSS: *r*(811) = −0.30 (−0.34 0.26), all *p* < 0.001) (Figure S5b).

Thus, lower IQ correlates with worse autistic symptoms, as shown by significant correlations between ELC and Total CSS scores (*r* around 0.2; consistent pattern for DQ and Total CSS: *r* around 0.3) for two different sets of children (longitudinal and singletons). These findings are significant because consistent patterns are detected for both sets of subjects, and because these patterns are based on continuous data. The patterns' direction is congruent with the main aim of the study ‐ linking autism manifestations with IQ. Given debates in the field about the intrinsically dichotomous way in which ASD diagnoses may be assigned at present (cut‐offs and clinical judgment), this correlation speaks to the idea that early childhood autism severity associates with important feature, IQ, in a continuous manner. Greater autistic impairments on the ADOS are associated with lower IQs.

### New questions probed and for future testing: Exceptional TD cases with low IQ, and ASD with high IQ

**FIGURE 5:**
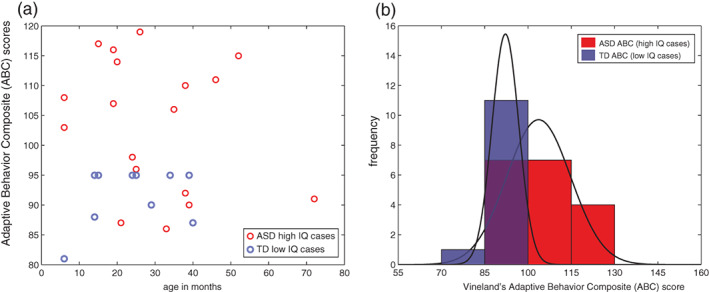
Intelligence quotient (IQ) and adaptive functioning for selected cases. (a) Scatterplot for ASD high IQ cases and TD low IQ cases, (b) same data, shown as frequency and corresponding probability density function (PDFs). Significantly worse adaptive functioning on the Vineland is detected for TD cases with full‐scale IQ (ELC) scores below 2SD, relative to ASD cases with ELC scores above 2SD (*p* < 0.05). ASD, Autism Spectrum Disorder; TD, typically developing; ABC, Adaptive Behavior Composite

Examination of Figures 1 and 3 reveals that there are exceptions to the pattern of ‘low IQ in ASD’ and ‘high IQ in TD’. There are some children with low IQ across all age time points who are not diagnosed as autistic and some children with high IQ who do have an ASD diagnosis. Here we asked if TD children with low IQ have some other problems, such as in adaptive functioning in daily life, focusing on the measure of adaptive functioning on the Vineland (Sparrow et al., 2005) since it was available for both groups. We detected worse adaptive functioning on the Vineland's Adaptive Behavior Composite (ABC) score for TD with lowest ELC scores, and improved adaptive functioning for ASD with higher ELC (*p* < 0.05) (Figure 5). Specifically, a Wilcoxon rank sum test indicated that the median ABC ranks for ASD, Mdn = 106.5, are significantly higher than the median *TD* ranks, Mdn = 95, *Z* = 2.62, *p* = 0.0086.

These outlier cases deserve a separate study to investigate further different types of hypotheses. Here we are framing this question from the ASD research perspective; it is possible that many of these would be children with ID, and not TD from the ID research perspective. However, it is important to note that with regard to low IQ TD children who are not diagnosed as autistic, none of the specific TD cases in Figure 5 have extremely low IQ (none score below 55, or below three SD on the ELC), and no known diagnoses (e.g., such as a learning disability). In a long‐term longitudinal population‐based study, we will track individual cases of early IQ, in particular, early high IQ ASD children. This approach will increase understanding about how early high IQ may compensate for social impairments, or even whether it may drive reduction of autism manifestations as these children grow.

One may note that some of the results presented may be novel to the field of ASD research, but may not be surprising in the field of ID research. Indeed, given the important role of adaptive behavior in the diagnosis of ID (see Introduction), one expects to observe worse adaptive functioning on the Vineland for children with the lowest ELC scores, and improved adaptive functioning for ASD with higher ELC scores. From the ASD research perspective, however, these results claim a new way to understand childhood autism, helping illuminate the strong link between cognitive abilities and daily functioning in early‐diagnosed children with ASD.

### New questions arising: is lower IQ in ASD related to an earlier age of diagnosis?

Considering the findings from the early infant assessments and childhood ASD diagnoses, in particular IQ assessments from 6 months of age, these data challenge the assumption that IQ is unimportant to ASD diagnosis, particularly for relatively early diagnosed children. The open question is whether low IQ precedes future ASD diagnosis in early childhood for some subset of the ASD population. For instance, a new study from the UK reports evidence on emerging, later‐diagnosed ASD cases (in adulthood) who have had low IQ in childhood, with measures obtained starting from 7 years of age (Riglin et al., 2021). Studies are needed to pursue this important new lead pertaining to the differential contribution of age at diagnosis and low IQ.

## DISCUSSION

This extremely large prospective study establishes that low IQ is an important feature of individuals diagnosed with ASD in early childhood. The core features of autism in this group are not independent of low IQ: before some of the children had ASD diagnoses, as infants, they had significantly lower cognitive abilities. Very early low IQ is a predictor of future ASD diagnosis made in early childhood. Early low IQ does not predispose all children to autism, since we detected a few of the low IQ cases who do not have ASD. However, low IQ can be considered as an early sign of abnormal brain development that leads to autism. These findings are based on the population involved in current autism research supported by the NIH in the US, and have important implications, considered as follows.

In this study, both verbal and non‐verbal IQ, as well as the estimate of overall, IQ or *g* (ELC, full‐scale IQ standard score) are significantly lower in the ASD group relative to TD from the start of life, and decline—however slightly—over time. Moreover, lower IQ significantly associates with worse autism symptoms in the ASD group. Importantly, *very early* (below 1 year of age) low IQ carries a higher risk ratio for future ASD diagnosis made by around 3–4 years. Thus, taken together, these findings strongly suggest an overall cognitive deficit that signals neurodevelopmental problems very early in life (below 1 year of age) for infants who later are diagnosed with an ASD.

### Why does low IQ characterize children diagnosed with ASD in early childhood?

An important concern in the field is that the current definition of autism (subsumed under the ASD label) may be “too vague” (Frith, 2021; Mottron, 2021) resulting in a diminished distinction between classic, or “prototypical” autism cases, and other types of neurodevelopmental disorders. Thus, one may ask whether the ASD group represents cases with a non‐specific neurodevelopmental disorder, with the ADOS mainly serving as an adjunct to ascertain difficulties in behavioral adaptation. Could results in our study be due to an overrepresentation of young children with low IQ in this ASD population, potentially due to over‐inclusive diagnostic criteria?

Despite the inherent challenges and difficulties associated with differential diagnoses, it is important to note that data in this study do not support the possibility of ‘over‐inclusive’ diagnoses in the ASD group. That is because cognitive abilities in the ASD group were significantly more impaired not only relative to the TD group, but also relative to the third group of subjects, for whom ASD has been ruled out but TD status not conferred (‘noASDdetected’: Figure S3, Table S6). If the ASD group represented mostly cases with a non‐specific neurodevelopmental disorder, then cognitive abilities of this separate, ‘noASDdetected’ group would be statistically similar to the ASD group, but in fact they are not similar (e.g., on the ELC). Not only were ASD group's cognitive abilities significantly worse relative to TD group, which is the main comparison group in this work, but also children with ASD were significantly worse relative to the group of children without ASD from the ‘noASDdetected’ group (who are not ascertained as TD, and for whom another neurodevelopmental disorder was not ruled out).

Future work is required to parse the phenotypic heterogeneity within the ‘noASDdetected’ group with regard to non‐ASD impairments vis‐à‐vis their higher IQ scores relative to the TD children. For example, it would be interesting to understand if the group's somewhat higher IQ relative to TD (while at the same time lower IQ relative to ASD) supported more normative functioning as children's development unfolded, thereby precluding the diagnosis of ASD.

According to the early formulation of autism, individuals with autism represent a subgroup within a low IQ population, and this subgroup may be characterized by a specific cognitive deficit of information processing (Frith, 1989), in particular, the lack of mentalizing capacity (Frith, 1992). Our findings are overall consistent with the idea that low IQ is a co‐occurring feature in ASD, but specifically when ASD is diagnosed in childhood. It could well be that within our low IQ ASD childhood‐diagnosed group there are cases of classic autism with a more specific cognitive deficit (i.e., weak central coherence; Frith, 1991), or perhaps of a different, developmentally‐specific nature, as yet to be determined. What is the nature of a cognitive impairment that is both specific and predictive of future ASD in infants, within the 1st year of life? This exciting and significant question has not yet been asked empirically in infants—and cannot be resolved at present with these data—requiring future work during the infancy period.

### Uncovering the basis of low IQ, early diagnosed ASD children

In this study, the question of whether low IQ is a secondary effect of some other problem cannot be answered at present and is left open. Indeed, one needs to be cautious when considering potential underlying mechanisms that may cause or contribute to these early differences in human behaviors. Here, we have a significant puzzle because it is impossible to make conclusions about the causal direction. An early brain dysfunction in children that is causing the core autistic features may directly or indirectly compromise performance on IQ tests in early life, and some factor (e.g., a genetic abnormality) which compromises this brain function may also affect the core features, directly or indirectly.

The early low IQ scores in early diagnosed children in this study may be an indicator of the impaired integrity of nascent neurodevelopmental function or structure. Again, these children may differ in etiology from individuals who receive diagnoses in adolescence or adulthood and whose IQ scores are in the average range. For this reason, it may not be advised to combine these subgroups for genetic or even neuroanatomical, brain imaging studies, since these children might differ in other (biological) respects as well, and this question should be a focus of future work. For instance, the molecular mechanisms of pathophysiology might differ, a possibility with direct implications for brain and behavior and for therapeutic interventions. The early diagnosed individuals (vs. later diagnosed children) may need different clinical management and treatment. It would be important to conduct in‐vivo, non‐invasive brain imaging studies to specifically compare brain circuitries in the two putative subgroups (early vs. later diagnosed).

Only a handful of studies investigated the brain basis of low IQ in ASD using Magnetic Resonance Imaging (MRI), in part because it is challenging to acquire scans with low IQ or low functioning individuals and children with neurodevelopmental disabilities including ASD, because of poor tolerability for the MRI environment. While challenging, it is indeed possible to use established, children‐ and family‐friendly MRI data acquisition protocols and to acquire good quality, low‐motion scans with school‐aged children with ASD while awake (Denisova et al., 2013), as well as while awake or asleep in preschool children (Lee et al., 2021), without sedation.

In particular, a recent MRI study of children followed longitudinally since age 2 included participants with megalencephaly (enlarged head size relative to height), with all children ascertained for ASD or typical development (Lee et al., 2021). In children diagnosed with ASD, large brains as indicated by structural MRI in early childhood remain large later, with the findings significantly different relative to TD children, for the megalencephaly group with ASD who also had lower IQ (Lee et al., 2021). In a study of resting‐state fMRI of children with ASD and low verbal and non‐verbal performance (vs. TD), Gabrielsen et al. (2018) detected reduced brain connectivity in low‐IQ ASD vs. higher‐IQ ASD and TD children and adolescents (Gabrielsen et al., 2018). Despite the challenges of acquiring MRI data in children, it is very important to include individuals with low IQ in brain imaging studies in order to be able to generalize MRI findings to all individuals with ASD (Lee et al., 2021). To address new questions raised in the current study, new MRI studies are required to examine brain structure and function of infants with different IQ levels during very early childhood from the general population and who are followed up for autism manifestations as they grow.

### Strengths and limitations

While we did not detect a sex difference in IQ at 6 months (i.e., combined girls and boys low IQ resulted in an elevated risk ratio for future ASD diagnosis, as well as in a males‐only group), the lack of female‐specific finding may be due to a small number of female infants available at 6 months. This question should be investigated in future work with a larger sample of female infants. The MSEL has been a common instrument to assess early developmental and cognitive abilities in the ASD field for many ASD research studies, including the early NIH's CPEA and current ACE projects. While this continuity with a single instrument by the field is what enabled the current project's extremely large dataset, the norms have not been updated in several decades. It would be important to collect new data and to update the Mullen norms in order to reflect the population of infants and children currently growing up in the US. However, in the current study, the normed scores on the Mullen were significantly correlated with scores on DAS‐II, an instrument with more recently updated norms. Alternatively, the Bayley scales measure permits characterization of domain‐based abilities, including for cognition, language, and motor skills (with norms calculated more recently).

All children were enrolled either as at‐risk infants (due to parental or other concerns, such as family history) or as age‐matched healthy controls in Autism Centers of Excellence or similar research centers and networks, or as participants in studies investigating normative development, in the US. It is possible that in this study, the children who were ascertained as typically developing may not necessarily represent the “normative” population at large, as these families may differ in some ways to those families unable to enroll their children (for example, proximity to an urban center, financial incentive to participate, or incentive to receive feedback on their children's functioning). However, in the current study, the ASD population was precisely ascertained: young children were recruited as part of the rigorously peer‐reviewed research funded by the NIH in the US, with DSM‐5 diagnoses supplemented with direct observational measures (the ADOS) of each child's behavior.

Importantly, by using direct observation information as part of the ascertainment, this study revealed that children diagnosed with ASD in childhood have significantly lower IQ in infancy, relative to those not only ascertained as being typically developing, normal children, but also relative to another set of children who are not ascertained to have ASD and are not ascertained as TD (and who may have a non‐ASD developmental disorder). In new studies, it would further be extremely important to ensure a more diverse and equitable recruitment of families, including those not necessarily within reach of regular recruitment efforts or regular ‘catchment’ areas, to gather an even more generalizable sample that reflects population diversity.

An important area of investigation for future work would be to develop a more precise, theoretically‐driven set of phenotypic criteria for autism manifestations in early childhood, while taking into account the likelihood for a neurobiological or genetic deficit at an individual level. Are there phenotypic metamers of early childhood autism, that is, cases that are phenotypically similar but in which different underlying (neuro)biological factors converge to produce autism manifestations in childhood?

## CONCLUSION

This extremely large study investigated systematically the role of individual differences in intelligence in early life vis‐à‐vis future ASD outcomes in childhood, and reflects the population with ASD involved in research studies currently funded by the NIH in the US. The key discovery is that low early IQ is a major feature of ASD that can be assessed objectively, reliably and early. The current findings raise important new questions on ASD diagnosed in early childhood, including on whether early‐diagnosed ASD cases may have different etiology and pathophysiology relative to those later diagnosed. Future work is required to investigate the specific and developmentally unique brain‐based and genetic mechanisms underlying the finding of low early IQ in ASD. This study is the first to establish prospectively that in children who go on to have ASD in childhood, cognitive abilities are already low starting from early infancy.

## CONFLICT OF INTEREST

The authors declare no conflict of interest with regard to the funding sources for this study.

## Supporting information


**Table S1**. The breakdown of data by NDA/NDAR collection number (i.e. submitter(s) identifier) and study site, reflecting sources across the United States (from at least 18 US states, and across‐state networks). The ordering reflects the number of subjects from a given collection (from high to low). *Note*: the overall total does not add up to 8,065 (the number of unique subjects across all collections with 15,030 assessments on the Mullen Scales of Early Learning) because some subjects had longitudinal assessments that continued as part of a different collection under a new grant. This overlap may reflect new initiatives by the original investigators as well as data sharing initiatives across investigators. The within‐collection totals for the 62 study‐sites (shown in decreasing number of unique subjects per site) are provided here for information only, in accordance with the requirements set by the NDA/NDAR Data Access/Sharing Agreement.
**Table S2**. Subject characteristics for the overall sample and for the longitudinal subset. The overall sample subsumes N = 8,065 unique children, including N = 6,029 with ascertained outcomes (ASD, TD, as well as those children for whom ASD was ruled out (‘noASDdetected’ subgroup). Note that the subsets with ascertained outcomes do not include datasets recruited as part of the Tuberous Sclerosis study (collection #2008). Information for subjects with ascertained outcomes, excluding the small number of datasets recruited as part of the Fragile‐X (“*Fg*‐*X*”) study (collection #1888), is also presented.
**Table S3**. Calibrated Severity Scores: SA, RRB, and total CSS. The scores are based on the revised algorithms of the ADOS‐G or ADOS‐2, and use the appropriate language level‐, age‐, and module‐specific mapping algorithms to convert the ADOS Raw Totals to CSS, for subjects for whom these scores could be computed (totaling N = 5,964; about 99% of the subjects with ascertained outcomes). SA: Social Affect, SA; RRB: Restricted and Repetitive Behavior, RRB.
**Table S4**. Parameter estimates from the linear multilevel model of age and IQ for the overall sample, regardless of availability of clinical outcomes. Results are presented separately for models with VIQ, PIQ, DQ, and IQ (ELC) as predictors. The ELC is the standard composite score on the Mullen, providing an estimate of IQ or g. Note: the ELC scores were not available for a small portion of the sample.
**Table S5**. Parameter estimates from the linear multilevel model of age and DQ for the ASD and TD.
**Table S6**. Parameter estimates from the linear multilevel model of age and VIQ, as well separate models of age and PIQ, age and DQ, and age and IQ (ELC)), for the noASDdetected group.
**Table S7**. Parameter estimates from the linear multilevel model of age and VIQ, as well separate models of age and PIQ, age and DQ, and age and IQ (ELC)), for the noASDdetected group, excluding Fragile‐X datasets (collection #1888).
**Table S8**. Parameter estimates from the linear multilevel model of age and VIQ, as well separate models of age and PIQ, age and DQ, and age and IQ (ELC)), for the ASD group, excluding Fragile‐X datasets (collection #1888).
**Figure S1**. Correlations between IQ scores at 6 mo and later time points at 12, 18, 24, 36 mo (shown for VIQ, PIQ, and ELC) (ELC, overall set: all *p* < 0.01). Note: correlations remain significant for the lowest scores (below median) at 6 mo on the ELC, with scores at 12, 18, 24, as well as 36 months.
**Figure S2**. Low IQ at 6 mo for both males and female infants who later received ASD diagnoses. The IQ (ELC) at 6 mo was significantly lower than the population mean of 100 for both males (p = 2.9948e‐11) and females (p = 7.2749e‐05); no difference in scores was detected between males and females (*p* > 0.05).
**Figure S3**. Linear multilevel models with age as predictor, for overall sample (a) and by subgroupings: ASD and TD are shown in (b), and ASD, TD, and ASD ruled out (‘noASDdetected’) are shown in (c). For all subgroupings, separate models were ran with response variables VIQ, PIQ, DQ, and ELC. Individual children were modeled as random effects.
**Figure S4**. IQ by age groupings: age bins and frequency histograms for infants with ASD and TD outcomes, who have one and only one Mullen assessment falling into one of the pre‐defined age bins. (a) Age bins (6, 12, 18, 24, 30, 36mo +/−1 mo). (b) For each of the age bins in (a), frequency and probability density function (PDFs) are shown. The width of the bars is 15, equal to the standard deviation of the Early Learning Composite (ELC) standard score.
**Figure S5**. Lower IQ (Age‐Equivalency‐based DQ scores) significantly associates with worse autism symptoms. DQ vs Social Affect (SA), Repetitive and Restricted Behaviors (RRB) and Total Calibrated Severity Scores (CSS) on the ADOS. Same Ns as in Figure 4 in the main text: (a) from infants with at least 2 Mullen assessments and (b) from a separate set of infants with one and only 1 assessment on the Mullen who were ascertained with ASD. DQ: Developmental Quotient.

## Data Availability

The data that support the findings of this study are openly available in NIMH Data Archive (NDA) at doi: 10.15154/1528140.
