# Supplementary material for: The importance of low IQ to early diagnosis of autism
Source: Autism Res. 2022 Nov 13;16(1):122–42. doi: 10.1002/aur.2842 (PMC9839551; doi:10.1002/aur.2842)
Supplement: Supplementary file 1 — Table S1. The breakdown of data by NDA/NDAR collection number (i.e. submitter(s) identifier) and study site, reflecting sources across the United States (from at least 18 US states, and across‐state networks). The ordering reflects the number of subjects from a given collection (from high to low). Note: the overall total does not add up to 8,065 (the number of unique subjects across all collections with 15,030 assessments on the Mullen Scales of Early Learning) because some subjects had longitudinal assessments that continued as part of a different collection under a new grant. This overlap may reflect new initiatives by the original investigators as well as data sharing initiatives across investigators. The within‐collection totals for the 62 study‐sites (shown in decreasing number of unique subjects per site) are provided here for information only, in accordance with the requirements set by the NDA/NDAR Data Access/Sharing Agreement. Table S2. Subject characteristics for the overall sample and for the longitudinal subset. The overall sample subsumes N = 8,065 unique children, including N = 6,029 with ascertained outcomes (ASD, TD, as well as those children for whom ASD was ruled out (‘noASDdetected’ subgroup). Note that the subsets with ascertained outcomes do not include datasets recruited as part of the Tuberous Sclerosis study (collection #2008). Information for subjects with ascertained outcomes, excluding the small number of datasets recruited as part of the Fragile‐X (“Fg‐X”) study (collection #1888), is also presented. Table S3. Calibrated Severity Scores: SA, RRB, and total CSS. The scores are based on the revised algorithms of the ADOS‐G or ADOS‐2, and use the appropriate language level‐, age‐, and module‐specific mapping algorithms to convert the ADOS Raw Totals to CSS, for subjects for whom these scores could be computed (totaling N = 5,964; about 99% of the subjects with ascertained outcomes). SA: Social Affect, SA; RRB: Restricted and Repetitive Behav [file AUR-16-122-s001.docx]

**Supporting Information**

**Supplementary Tables**

| **Order** | **N subjects** | **N assessments** | **NDA/NDAR collection # (submitter(s) identifier, and Title)** | **Principal Investigator(s)** | **Location of main PI (w/ US state)** |
| --- | --- | --- | --- | --- | --- |
| 1 | 783 | 1127 | [2115] Detection of ASD at the 1st birthday as standard of care: The Get SET Early Model | Karen Pierce | University of California, San Diego (UCSD ACE) (CA) |
| 2 | 782 | 1543 | [9] Biomarkers of Autism at 12 Months: From Brain Overgrowth to Genes | Eric Courchesne, Karen Pierce and others | University of California, San Diego (UCSD ACE) (CA) |
| 3 | 491 | 1281 | [19] Longitudinal MRI Study of Infants at Risk for Autism | Joe Piven | University of North Carolina, Chapel Hill (NC) |
| 4 | 452 | 452 | [2066] The CHARGE Study: Childhood Autism Risks From Genetics and the Environment | Irva Hertz-Picciotto | University of California, Davis (UC-Davis) (CA) |
| 5 | 421 | 727 | [2384] Early Brain Development in Twins | John Gilmore | University of North Carolina, Chapel Hill (NC) |
| 6 | 390 | 775 | [1250] Screening and Diagnostic Measures in Toddlers with ASD | Amy Wetherby | Florida State University (FL) |
| 7 | 358 | 379 | [2666] Early Detection of Autism Spectrum Disorder | Diana Robins | Drexel University / NICHD (PA) |
| 8 | 323 | 509 | [2080] Smart Early Screening for Autism and Communication Disorders in Primary Care | Amy Wetherby | Florida State University (FL) |
| 9 | 259 | 794 | [1946] Autism Risk, Prenatal Environmental Exposures, and Pathophysiologic Markers | Irva Hertz-Picciotto | University of California, Davis (UC-Davis) (CA) |
| 10 | 241 | 605 | [1600] Early Autism Risk Longitudinal Investigation (EARLI) Network | Craig Newschaffer | Drexel University / EARLI Network (PA) |
| 11 | 229 | 432 | [2028] Mechanisms of Risk and Resilience in ASD: Ontogeny, Phylogeny and Gene Disruption | Amy Klin | Emory University (GA) |
| 12 | 222 | 341 | [2293] Components of Emotional Processing in Toddlers with ASD | Katarzyna Chawarska | Yale University (CT) |
| 13 | 204 | 222 | [1886] PDN Screening Data | Audrey Thurm | Intramural NIH Investigator (MD) |
| 14 | 194 | 301 | [8] Yale Autism Center of Excellence: Mechanisms of Social Engagement in the Autism Spectrum Disorders | Fred Volkmar | Yale University (CT) |
| 15 | 189 | 315 | [1654] UW ACE Extended Family Study | Bryan King | University of Washington (UW ACE) (WA) |
| 16 | 183 | 434 | [2368] Clinical and Immunological Investigations of Subtypes of Autism | Susan Swedo | Intramural NIH Investigator (MD) |
| 17 | 177 | 177 | [2113] Addressing systemic health disparities in early ASD identification and treatment | Alice Carter | University of Massachusetts (Boston) (MA) |
| 18 | 176 | 519 | [2026] Biomarkers of Developmental Trajectories and Treatment in ASD | Susan Bookheimer | University of California, Los Angeles (UCLA ACE) (CA) |
| 19 | 136 | 165 | [2290] Early Identification of ASD: Translating Eye Tracking into Practice (Years 6-10) | Karen Pierce | University of California, San Diego (UCSD ACE) (CA) |
| 20 | 158 | 849 | [2008] Early Biomarkers of Autism Spectrum Disorders in infants with Tuberous Sclerosis | Mustafa Sahin | Harvard University (Boston) (MA) |
| 21 | 146 | 494 | [2024] Divergent biases for conspecifics as early markers for Autism Spectum Disorders | Athena Vouloumanos | New York University (NY) |
| 22 | 145 | 145 | [14] Early Pharmacotherapy Guided by Biomarkers in Autism | Diane Chugani | Wayne State and Delaware University (DE) |
| 23 | 138 | 374 | [6] Biological and Information Processing Mechanisms Underlying Autism | Nancy Minshew; multiple PIs | Pittsburg University (PA) |
| 24 | 136 | 136 | [1358] Biological Signatures of Autism | David Amaral | University of California, Davis (UC-Davis) (CA) |
| 25 | 128 | 349 | [1888] Emergence and Stability of Autism in Fragile X Syndrome | Jane Roberts | University of South Carolina (SC) |
| 26 | 129 | 188 | [2557] Environmental Influence on Infant Microbiome Development and ASD Symptoms | Irva Hertz-Picciotto | University of California, Davis (UC-Davis) (CA) |
| 27 | 98 | 98 | [16] Intensive Intervention for Toddlers With Autism (EARLY STEPS) | Sally Rogers | University of California, Davis (UC-Davis) (CA) |
| 28 | 88 | 88 | [2036] Intervention effects of intensity and delivery style for toddlers with ASD | Sally Rogers | University of California, Davis (UC-Davis) (CA) |
| 29 | 84 | 84 | [10] UCLA Sigman/Bookheimer ACE and ARRA | Marian Sigman, Susan Bookheimer | University of California, Los Angeles (UCLA ACE) (CA) |
| 30 | 83 | 83 | [2108] Neural Phenotypes of Females with Autism Spectrum Disorder | Christine Nordahl | University of California, Davis (UC-Davis) (CA) |
| 31 | 78 | 266 | [2027] A Longitudinal MRI Study of Infants at Risk for Autism (ACE 2) | Joe Piven | University of North Carolina, Chapel Hill (NC) |
| 32 | 76 | 80 | [2077] Gaze Modification Strategies for Toddlers with ASD | Frederic Shic, Katarzyna Chawarska | Yale University / University of Washington (Seattle) (WA) |
| 33 | 55 | 56 | [2262] Comparing Behavioral Assessments Using Telehealth for Children with Autism | Scott David Lindgren | University of Iowa (IA) |
| 34 | 49 | 49 | [2761] Charting the trajectory of executive control in autism in order to optimize delivery of intervention | Susan Faja | Boston Children's Hospital (MA) |
| 35 | 48 | 48 | [1866] 1/2 Development of a Screening Interview for Research Studies of ASD | Christopher Monk, Somer Bishop, Cathy Lord | University of Michigan (MI) |
| 36 | 45 | 45 | [1885] Predicting Useful Speech in Children With Autism | Paul Yoder | Vanderbilt University (TN) |
| 37 | 36 | 36 | [2004] Sequencing Autism Spectrum Disorder Extended Pedigrees (1/3, 2/3, and 3/3) | Gerard D. Schellenberg, Hilary Coon, Ellen M. Wijsman | University of Pennsylvania School of Medicine (PA) |
| 38 | 35 | 124 | [1920] Visual attention and fine motor coordination in infants at risk for autism | Anjana Bhat | University of Connecticut (CT) |
| 39 | 35 | 35 | [1923] Components of Limited Activity Monitoring in Toddlers with ASD | Frederic Shic | Yale University / University of Washington (Seattle) (WA) |
| 40 | 30 | 30 | [1300] Behavioral Treatment for Autism in Community Settings Using a Telehealth Network | Scott Lindgren | University of Iowa (IA) |
| 41 | 30 | 35 | [2183] Identifying Biomarkers for Early Detection of Prosody Disorders in ASD using Electroglottography | Shweta Ghai, Gordon Ramsay | Emory University (GA) |
| 42 | 24 | 24 | [2338] Multimodal Imaging of Early Neural Signature in Autism Spectrum Disorder | Inna Fishman | San Diego State University (CA) |
| 43 | 21 | 21 | [1] UIC ACE: Translational Studies of Insistence on Sameness in Autism | Ed Cook | University of Illinois at Chicago (UIC ACE) (IL) |
| 44 | 19 | 19 | [2495] Functions and Development of the Mirror Neuron System | Nathan Fox | University of Maryland, College Park (MD) |
| 45 | 19 | 19 | [2878] Social Rhythmic Entrainment and Language Development in Autism Spectrum Disorders | Miriam Lense | Vanderbilt University Medical Center (TN) |
| 46 | 15 | 15 | [2702] The Autism MEAL Plan: A Parent Training Curriculum to Manage Eating Aversions and Limited Variety among Children with Autism | William Sharp | Emory University (GA) |
| 47 | 17 | 17 | [2777] Center for the Development of Phenotype-Based Treatments of Autism Spectrum Disorder | David Amaral | University of California, Davis (UC-Davis) (CA) |
| 48 | 15 | 28 | [2009] Study of Oxytocin in Autism to Improve Reciprocal Social Behaviors (SOARS-B) | Linmarie Sikich | Duke University (ACE SOARS) (NC) |
| 49 | 13 | 13 | [2012] Optimization of Fidelity Procedures for Pivotal Response Training in Autism | Aubyn Stahmer | University of California, San Diego (UCSD) (CA) |
| 50 | 12 | 12 | [2349] RUPP PI PDD: Drug and Behavioral Therapy for Children With Pervasive Developmental Disorders | Michael Aman | Ohio State University (OH) |
| 51 | 10 | 10 | [2556] Behavioral Economic Measures of Sensitivity to Social Reward in Children with ASD | Nathan Call | Emory University (GA) |
| 52 | 10 | 10 | [2005] Pivotal Response Treatment for Infants at Risk for ASD: A Pilot Intervention | Katarzyna Chawarska | Yale University (CT) |
| 53 | 8 | 8 | [1854] Studies to Advance Autism Research and Treatment (STAART). | The STAART Network | The STAART Network (eight centers across the country) (n/a) |
| 54 | 8 | 8 | [2355] Neurobehavioral Research on Infants at Risk for Language Delay and ASD | Helen Tager-Flusberg | Boston University (MA) |
| 55 | 9 | 12 | [2774] Improving Child-Treatment Fit in Autism Early Intervention | Giacomo Vivanti | Drexel University (PA) |
| 56 | 4 | 4 | [2503] A new treatment for minimally verbal girls with ASD | Tom Cariveau | University of North Carolina, Wilmington (NC) |
| 57 | 3 | 8 | [1800] Initial Investigation of Prevention of ASD in Infants at Risk | Sally Rogers | University of California, Davis (UC-Davis) (CA) |
| 58 | 4 | 4 | [2599] A Multimedia Screening System for Early ASD Identification in Diverse Populations | Katarzyna Chawarska | Yale University (CT) |
| 59 | 3 | 3 | [1906] Atypical Late Neurodevelopment in Autism | Janet E Lainhart | University of Wisconsin-Madison (WI) |
| 60 | 3 | 3 | [2821] Cellular, molecular, and functional imaging approaches to understanding early neurodevelopment in autism | Katarzyna Chawarska | Yale University (CT) |
| 61 | 1 | 1 | [1201] Induced pluripotent cells in ASD with macrocephaly | Flora Vaccarino | Yale University (CT) |
| 62 | 1 | 1 | [2025] Minimally Verbal ASD: From Basic Mechanisms to Innovative Interventions | Helen Tager-Flusberg | Boston University ACE (MA) |
| Total | 8279 | **15030** |  |  |  |

**Table S1.** The breakdown of data by NDA/NDAR collection number (i.e. submitter(s) identifier) and study site, reflecting sources across the United States (from at least 18 US states, and across-state networks). The ordering reflects the number of subjects from a given collection (from high to low). *Note*: the overall total does not add up to 8,065 (the number of unique subjects across all collections with 15,030 assessments on the Mullen Scales of Early Learning) because some subjects had longitudinal assessments that continued as part of a different collection under a new grant. This overlap may reflect new initiatives by the original investigators as well as data sharing initiatives across investigators. The within-collection totals for the 62 study-sites (shown in decreasing number of unique subjects per site) are provided here for information only, in accordance with the requirements set by the NDA/NDAR Data Access/Sharing Agreement.

| All subjects | | |
| --- | --- | --- |
|  | Overall sample | Longitudinal (3 or more) |
| All children (total N) | 8,065 | 1,956 |
| Assessments (total) | 15,030 | 7,052 |
| Sex (m/f) | 5,485 m / 2,580 f | 1,236 m / 720 f |
| Age (median; months) | 23 | 18 |
| Age (range (min, max; months)) | 2 - 68 | 2 - 68 |
|  |  |  |
| Subset of subjects with ascertained outcomes | | |
|  | Overall | Longitudinal (3 or more) |
| ASD (total N) | 3,098 | 535 |
| Assessments (total) | 5,036 | 1,828 |
| Sex (m/f) | 2,439 m / 659 f | 418 m / 117 f |
| Age (median; months) | 27 | 24 |
| Age (range (min, max; months)) | 3 - 68 | 3 - 68 |
|  |  |  |
| TD (total N) | 691 | 215 |
| Assessments (total) | 1,370 | 736 |
| Sex (m/f) | 412 m / 279 f | 127 m / 88 f |
| Age (median; months) | 22 | 24 |
| Age (range (min, max; months)) | 3 - 68 | 3 - 68 |
|  |  |  |
| ASD ruled out (‘noASDdetected’) (total N) | 2,240 | 638 |
| Assessments (total) | 4,492 | 2,297 |
| Sex (m/f) | 1,413 m / 827 f | 375 m / 263 f |
| Age (median; months) | 22 | 19 |
| Age (range (min, max; months)) | 3 - 68 | 3 - 67 |
|  |  |  |
| Subset of subjects with ascertained outcomes (without Fragile-X collection) | | |
|  | Overall | Longitudinal (3 or more) |
| ASD (total N) (*no Fg-X*) | 3,050 | 508 |
| Assessments (total) | 4,916 | 1,739 |
| Sex (m/f) | 2,398 m / 652 f | 393 m / 115 f |
| Age (median; months) | 28 | 24 |
| Age (range (min, max; months)) | 3 - 68 | 3 - 68 |
|  |  |  |
| TD (total N) (*no change - no Fg-X*) | 691 | 215 |
| Assessments (total) | 1,370 | 736 |
| Sex (m/f) | 412 m / 279 f | 127 m / 88 f |
| Age (median; months) | 22 | 24 |
| Age (range (min, max; months)) | 3 - 68 | 3 - 68 |
|  |  |  |
| ASD ruled out (‘noASDdetected’) (total N) (*no Fg-X*) | 2,168 | 582 |
| Assessments (total) | 4,278 | 2,107 |
| Sex (m/f) | 1,373 m / 795 f | 341 m / 241 f |
| Age (median; months) | 22 | 19 |
| Age (range (min, max; months)) | 3 - 68 | 3 - 67 |

**Table S2.** Subject characteristics for the overall sample and for the longitudinal subset. The overall sample subsumes N=8,065 unique children, including N=6,029 with ascertained outcomes (ASD, TD, as well as those children for whom ASD was ruled out (‘noASDdetected’ subgroup). Note that the subsets with ascertained outcomes do not include datasets recruited as part of the Tuberous Sclerosis study (collection #2008). Information for subjects with ascertained outcomes, excluding the small number of datasets recruited as part of the Fragile-X (“*Fg-X”*) study (collection #1888), is also presented.

| Overall sample (ascertained outcomes w/ available scores) | | | | Longitudinal subset | | | |
| --- | --- | --- | --- | --- | --- | --- | --- |
|  | SA CSS  (mean, +/- sd) | RRB CSS  (mean, +/- sd) | Total CSS  (mean, +/- sd) |  | SA CSS  (mean, +/- sd) | RRB CSS  (mean, +/- sd) | Total CSS  (mean, +/- sd) |
| ASD  (N=3,045) | 6.74 (2.08) | 7.15 (2.21) | 6.86 (2.05) | ASD  (N=516) | 6.25 (1.89) | 6.78 (2.46) | 6.30 (1.93) |
| TD  (N=691) | 1.59 (0.90) | 2.29 (1.99) | 1.47 (0.76) | TD  (N=215) | 1.43 (0.73) | 2.19 (1.97) | 1.16 (0.44) |
| ASD ruled out (‘noASDdetected’) (N=2,228) | 2.25 (1.42) | 3.32 (2.40) | 1.98 (1.27) | ASD  ruled out (N=636) | 1.97 (1.13) | 3.39 (2.38) | 1.63 (0.90) |

**Table S3.** Calibrated Severity Scores: SA, RRB, and total CSS. The scores are based on the revised algorithms of the ADOS-G or ADOS-2, and use the appropriate language level-, age-, and module-specific mapping algorithms to convert the ADOS Raw Totals to CSS, for subjects for whom these scores could be computed (totaling N=5,964; about 99% of the subjects with ascertained outcomes). SA: Social Affect, SA; RRB: Restricted and Repetitive Behavior, RRB.

Overall sample

VIQ

Fixed effects coefficients (age) parameter estimate se 95% CIs df t-value p-value

β_0_ intercept (grand-average) 84.99 0.43 84.13 85.85 15028 194.23 <0.001

β_1_ slope (grand-average) -0.17 0.01 -0.21 -0.14 15028 -10.484 1.2564e-25

Random effects covariance (child) parameter estimate 95% CIs

Intercept (st.dev) 16.51 15.33 17.78

age (st.dev) 0.65 0.60 0.70

correlation: age, intercept -0.03 -0.14 0.07

Observations 15030

R^2^ 0.76

Adj. R^2^ 0.76

F-statistic 109.91 (df=1, 15028), p=1.2564e-25

Resid. st.dev. 15.80 (15.46 16.16 95% CIs)

PIQ

Fixed effects coefficients (age) parameter estimate se 95% CIs df t-value p-value

β_0_ intercept (grand-average) 109.48 0.37 108.75 110.22 15028 291.36 <0.001

β_1_ slope (grand-average) -0.62 0.01 -0.65 -0.59 15028 -43.27 <0.001

Random effects covariance (child) parameter estimate 95% CIs

Intercept (st.dev) 15.27 14.45 16.14

age (st.dev) 0.59 0.55 0.62

correlation: age, intercept -0.47 -0.53 -0.41

Observations 15030

R^2^ 0.74

Adj. R^2^ 0.74

F-statistic 1872.3 (df=1, 15028), p<0.001

Resid. st.dev. 13.44 (13.185 13.70 95% CIs)

DQ

Fixed effects coefficients (age) parameter estimate se 95% CIs df t-value p-value

β_0_ intercept (grand-average) 96.15 0.35 95.45 96.84 15028 271.53 <0.001

β_1_ slope (grand-average) -0.36 0.01 -0.39 -0.33 15028 -25.68 2.2471e-142

Random effects covariance (child) parameter estimate 95% CIs

Intercept (st.dev) 14.75 13.91 15.64

age (st.dev) 0.61 0.58 0.65

correlation: age, intercept -0.17 -0.25 -0.09

Observations 15030

R^2^ 0.84

Adj. R^2^ 0.84

F-statistic 659.47 (df=1, 15028), p=2.2471e-142

Resid. st.dev. 11.66 (11.40 11.91 95% CIs)

ELC

Fixed effects coefficients (age) parameter estimate se 95% CIs df t-value p-value

β_0_ intercept (grand-average) 91.88 0.33 91.21 92.54 11119 271.62 <0.001

β_1_ slope (grand-average) -0.01 0.01 -0.03 0.02 11119 -0.40 0.68

Random effects covariance (child) parameter estimate 95% CIs

Intercept (st.dev) 9.96 8.98 11.04

age (st.dev) 0.49 0.45 0.53

correlation: age, intercept 0.03 -0.11 0.18

Observations 11121

R^2^ 0.72

Adj. R^2^ 0.72

F-statistic 0.16 (df=1, 11119), p=0.68195

Resid. st.dev. 11.25 (10.98 11.53 95% CIs)

**Table S4**. Parameter estimates from the linear multilevel model of age and IQ for the overall sample, regardless of availability of clinical outcomes. Results are presented separately for models with VIQ, PIQ, DQ, and IQ (ELC) as predictors. The ELC is the standard composite score on the Mullen, providing an estimate of IQ or *g*. Note: the ELC scores were not available for a small portion of the sample.

ASD

DQ

Fixed effects coefficients (age) parameter estimate se 95% CIs df t-value p-value

β_0_ intercept (grand-average) 81.99 0.74 80.53 83.45 5034 109.92 <0.001

β_1_ slope (grand-average) -0.41 0.02 -0.46 -0.36 5034 -17.398 7.0084e-66

Random effects covariance (child) parameter estimate 95% CIs

Intercept (st.dev) 19.33 17.66 21.15

age (st.dev) 0.61 0.55 0.67

correlation: age, intercept -0.54 -0.62 -0.46

Observations 5036

R^2^ 0.87

Adj. R^2^ 0.87

F-statistic 302.69 (df=1, 5034), p=7.0084e-66

Resid. st.dev. 11.06 (10.58 11.56 95% CIs)

TD

DQ

Fixed effects coefficients (age) parameter estimate se 95% CIs df t-value p-value

β_0_ intercept (grand-average) 104.18 0.90 102.4 105.96 1368 114.59 <0.001

β_1_ slope (grand-average) 0.05 0.03 -0.004 0.12 1368 1.82 0.068533

Random effects covariance (child) parameter estimate 95% CIs

Intercept (st.dev) 12.10 9.91 14.77

age (st.dev) 0.23 0.12 0.43

correlation: age, intercept -0.80 -0.91 -0.59

Observations 1370

R^2^ 0.35

Adj. R^2^ 0.35

F-statistic 3.32 (df=1, 1368), p=0.068533

Resid. st.dev. 10.47 (9.80 11.17 95% CIs)

**Table S5**. Parameter estimates from the linear multilevel model of age and DQ for the ASD and TD.

ASD ruled out (‘noASDdetected’)

VIQ

Fixed effects coefficients (age) parameter estimate se 95% CIs df t-value p-value

β_0_ intercept (grand-average) 80.91 0.71 79.51 82.31 4490 113.56 <0.001

β_1_ slope (grand-average) 0.33 0.02 0.28 0.39 4490 12.80 6.9808e-37

Random effects covariance (child) parameter estimate 95% CIs

Intercept (st.dev) 16.29 14.36 18.48

age (st.dev) 0.45 0.37 0.54

correlation: age, intercept -0.14 -0.35 0.08

Observations 4492

R^2^ 0.65

Adj. R^2^ 0.65

F-statistic 163.9 (df=1, 4490), p=6.9808e-37

Resid. st.dev. 14.66 (14.11 15.23 95% CIs)

PIQ

Fixed effects coefficients (age) parameter estimate se 95% CIs df t-value p-value

β_0_ intercept (grand-average) 107.65 0.60 106.47 108.83 4490 178.65 <0.001

β_1_ slope (grand-average) -0.29 0.02 -0.33 -0.24 4490 -11.894 3.8265e-32

Random effects covariance (child) parameter estimate 95% CIs

Intercept (st.dev) 12.46 10.96 14.16

age (st.dev) 0.50 0.44 0.57

correlation: age, intercept -0.51 -0.62 -0.39

Observations 4492

R^2^ 0.56

Adj. R^2^ 0.56

F-statistic 141.48 (df=1, 4490), p=3.8265e-32

Resid. st.dev. 12.88 (12.43 13.35 95% CIs)

DQ

Fixed effects coefficients (age) parameter estimate se 95% CIs df t-value p-value

β_0_ intercept (grand-average) 93.49 0.56 92.39 94.59 4490 165.98 <0.001

β_1_ slope (grand-average) 0.04 0.02 0.01 0.09 4490 2.25 0.024108

Random effects covariance (child) parameter estimate 95% CIs

Intercept (st.dev) 13.38 12 14.92

age (st.dev) 0.45 0.40 0.51

correlation: age, intercept -0.22 -0.37 -0.07

Observations 4492

R^2^ 0.74

Adj. R^2^ 0.74

F-statistic 5.09 (df=1, 4490), p=0.024108

Resid. st.dev. 10.91 (10.50 11.33 95% CIs)

ELC

Fixed effects coefficients (age) parameter estimate se 95% CIs df t-value p-value

β_0_ intercept (grand-average) 89.8 0.53 88.75 90.84 4144 168.08 <0.001

β_1_ slope (grand-average) 0.22 0.02 0.17 0.26 4144 9.86 1.0307e-22

Random effects covariance (child) parameter estimate 95% CIs

Intercept (st.dev) 10.02 8.41 11.94

age (st.dev) 0.42 0.36 0.49

correlation: age, intercept -0.02 -0.26 0.22

Observations 4146

R^2^ 0.65

Adj. R^2^ 0.65

F-statistic 97.35 (df=1, 4144), p=1.0307e-22

Resid. st.dev. 11.29 (10.84 11.76 95% CIs)

**Table S6**. Parameter estimates from the linear multilevel model of age and VIQ, as well separate models of age and PIQ, age and DQ, and age and IQ (ELC)), for the noASDdetected group.

ASD ruled out (‘noASDdetected’) (without Fragile-X datasets)

VIQ

Fixed effects coefficients (age) parameter estimate se 95% CIs df t-value p-value

β_0_ intercept (grand-average) 80.33 0.73 78.88 81.78 4276 108.73 <0.001

β_1_ slope (grand-average) 0.35 0.02 0.29 0.40 4276 12.87 2.9095e-37

Random effects covariance (child) parameter estimate 95% CIs

Intercept (st.dev) 16.71 14.70 18.99

age (st.dev) 0.46 0.38 0.55

correlation: age, intercept -0.18 -0.39 0.03

Observations 4278

R^2^ 0.66

Adj. R^2^ 0.66

F-statistic 165.85 (df=1, 4276), p=2.9095e-37

Resid. st.dev. 14.58 (14.58 15.17 95% CIs)

PIQ

Fixed effects coefficients (age) parameter estimate se 95% CIs df t-value p-value

β_0_ intercept (grand-average) 106.73 0.60 105.54 107.91 4276 176.81 <0.001

β_1_ slope (grand-average) -0.26 0.02 -0.31 -0.21 4276 -10.67 2.7206e-26

Random effects covariance (child) parameter estimate 95% CIs

Intercept (st.dev) 11.85 10.32 13.608

age (st.dev) 0.49 0.43 0.56

correlation: age, intercept -0.46 -0.58 -0.32

Observations 4278

R^2^ 0.58

Adj. R^2^ 0.58

F-statistic 114.05 (df=1, 4276), p=2.7206e-26

Resid. st.dev. 12.58 (12.12 13.06 95% CIs)

DQ

Fixed effects coefficients (age) parameter estimate se 95% CIs df t-value p-value

β_0_ intercept (grand-average) 92.78 0.57 91.65 93.91 4276 161.86 <0.001

β_1_ slope (grand-average) 0.06 0.02 0.02 0.11 4276 3.05 0.0022

Random effects covariance (child) parameter estimate 95% CIs

Intercept (st.dev) 13.21 11.79 14.81

age (st.dev) 0.45 0.40 0.51

correlation: age, intercept -0.20 -0.35 -0.04

Observations 4278

R^2^ 0.75

Adj. R^2^ 0.75

F-statistic 9.34 (df=1, 4276), p=0.0022

Resid. st.dev. 10.77 (10.35 11.21 95% CIs)

ELC

Fixed effects coefficients (age) parameter estimate se 95% CIs df t-value p-value

β_0_ intercept (grand-average) 89.2 0.54 88.17 90.31 3935 163.59 <0.001

β_1_ slope (grand-average) 0.23 0.02 0.18 0.27 3935 10.26 2.0987e-24

Random effects covariance (child) parameter estimate 95% CIs

Intercept (st.dev) 9.69 7.9793 11.769

age (st.dev) 0.42 0.36 0.49

correlation: age, intercept -0.02 -0.25 0.29

Observations 3937

R^2^ 0.65

Adj. R^2^ 0.65

F-statistic 105.33 (df=1, 3935), p=2.0987e-24

Resid. st.dev. 11.29 (10.84 11.76 95% CIs)

**Table S7**. Parameter estimates from the linear multilevel model of age and VIQ, as well separate models of age and PIQ, age and DQ, and age and IQ (ELC)), for the noASDdetected group, excluding Fragile-X datasets (collection #1888).

ASD (without Fragile-X datasets)

VIQ

Fixed effects coefficients (age) parameter estimate se 95% CIs df t-value p-value

β_0_ intercept (grand-average) 65.40 0.94 63.56 67.25 4914 69.42 <0.001

β_1_ slope (grand-average) -0.18 0.02 -0.24 -0.12 4914 -6.25 4.3461e-10

Random effects covariance (child) parameter estimate 95% CIs

Intercept (st.dev) 21.63 19.33 24.20

age (st.dev) 0.57 0.48 0.68

correlation: age, intercept -0.45 -0.57 -0.31

Observations 4916

R^2^ 0.76

Adj. R^2^ 0.76

F-statistic 39.11 (df=1, 4914), p=4.3461e-10

Resid. st.dev. 15.65 (14.96 16.38 95% CIs)

PIQ

Fixed effects coefficients (age) parameter estimate se 95% CIs df t-value p-value

β_0_ intercept (grand-average) 100.38 0.72 98.95 101.79 4914 138.71 <0.001

β_1_ slope (grand-average) -0.70 0.02 -0.74 -0.65 4914 -30.39 4.7908e-186

Random effects covariance (child) parameter estimate 95% CIs

Intercept (st.dev) 16.94 15.26 18.82

age (st.dev) 0.55 0.50 0.61

correlation: age, intercept -0.59 -0.68 -0.51

Observations 4916

R^2^ 0.81

Adj. R^2^ 0.81

F-statistic 923.53 (df=1, 4914), p=4.7908e-186

Resid. st.dev. 12.01 (11.54 12.49 95% CIs)

DQ

Fixed effects coefficients (age) parameter estimate se 95% CIs df t-value p-value

β_0_ intercept (grand-average) 81.65 0.75 80.17 83.13 4914 108.1 <0.001

β_1_ slope (grand-average) -0.41 0.02 -0.45 -0.36 4914 -16.87 3.5655e-62

Random effects covariance (child) parameter estimate 95% CIs

Intercept (st.dev) 19.11 17.41 20.98

age (st.dev) 0.61 0.55 0.67

correlation: age, intercept -0.54 -0.62 -0.45

Observations 4916

R^2^ 0.87

Adj. R^2^ 0.87

F-statistic 284.88 (df=1, 4914), p=3.5655e-62

Resid. st.dev. 11.05 (10.55 11.56 95% CIs)

ELC

Fixed effects coefficients (age) parameter estimate se 95% CIs df t-value p-value

β_0_ intercept (grand-average) 82.67 0.80 81.08 84.25 2839 102.3 <0.001

β_1_ slope (grand-average) -0.10 0.02 -0.15 -0.04 2839 -3.48 <0.001

Random effects covariance (child) parameter estimate 95% CIs

Intercept (st.dev) 12.90 10.68 15.57

age (st.dev) 0.41 0.33 0.51

correlation: age, intercept -0.37 -0.57 -0.12

Observations 2841

R^2^ 0.66

Adj. R^2^ 0.66

F-statistic 12.12 (df=1, 2839), p=0.00050422

Resid. st.dev. 11.63 (11.01 12.27 95% CIs)

**Table S8**. Parameter estimates from the linear multilevel model of age and VIQ, as well separate models of age and PIQ, age and DQ, and age and IQ (ELC)), for the ASD group, excluding Fragile-X datasets (collection #1888).

**Supplementary Figures**


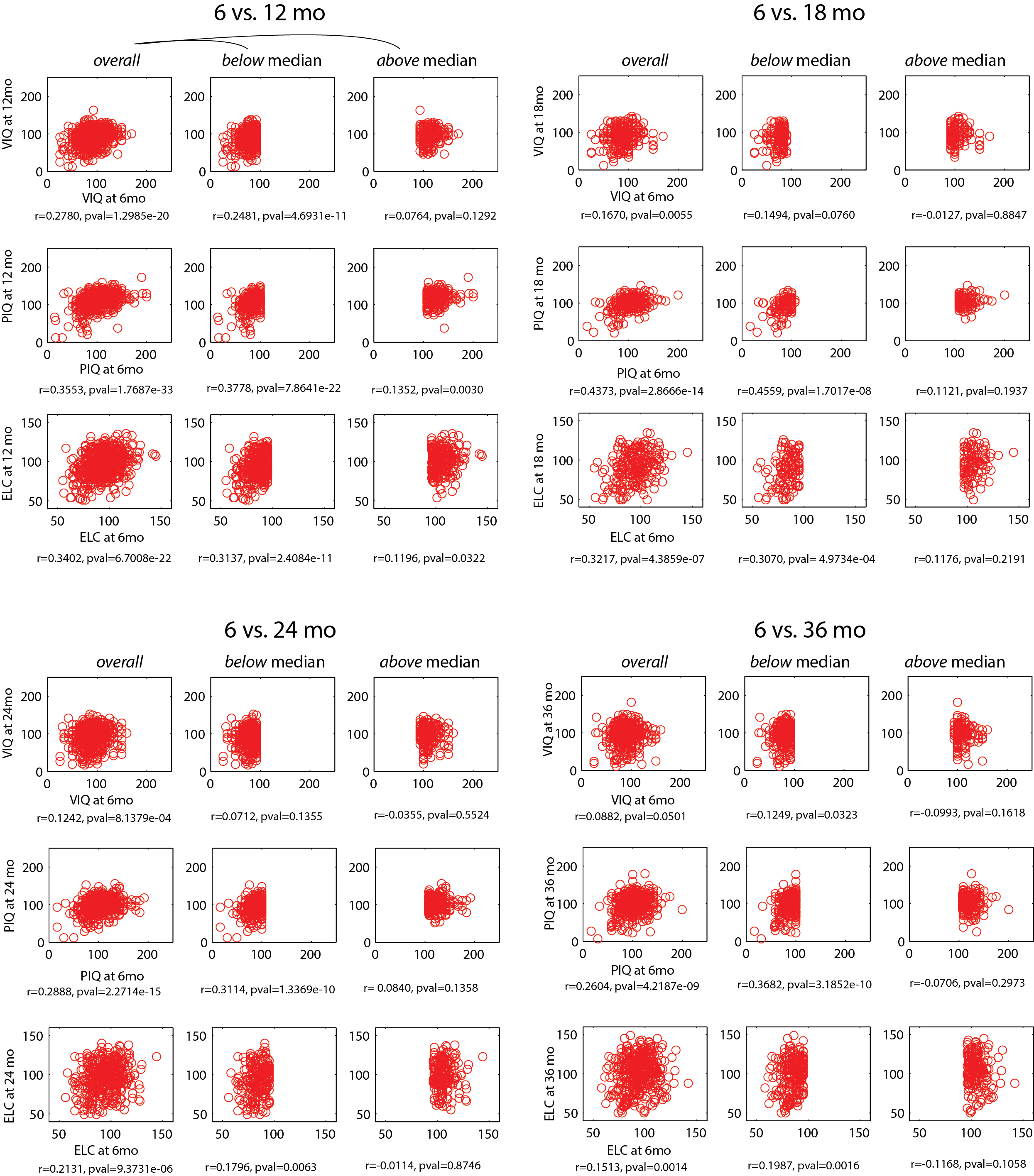


**Figure S1**. Correlations between IQ scores at 6 mo and later time points at 12, 18, 24, 36 mo (shown for VIQ, PIQ, and ELC) (ELC, overall set: all p<0.01). Note: correlations remain significant for the lowest scores (below median) at 6 mo on the ELC, with scores at 12, 18, 24, as well as 36 months.

**
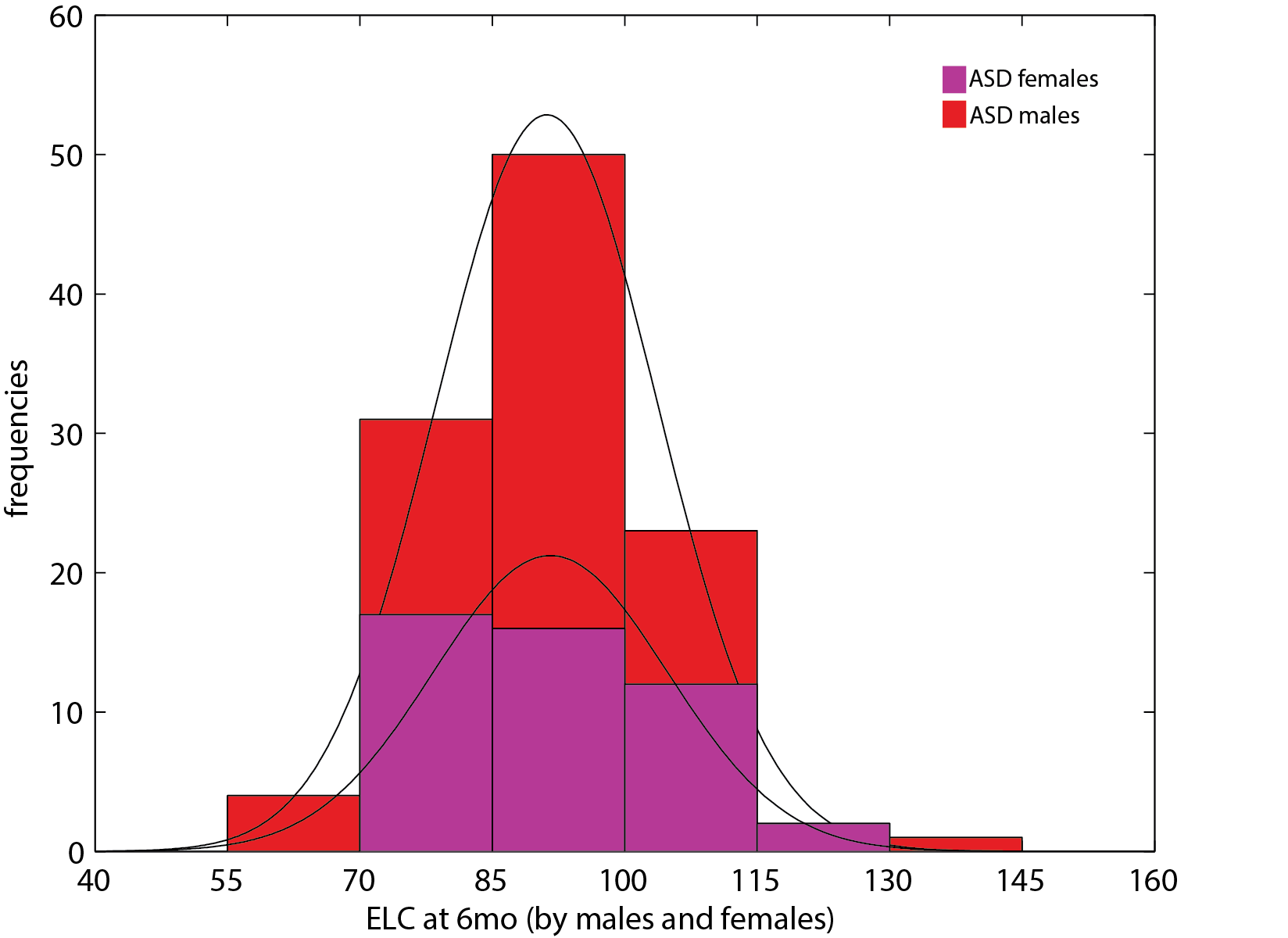
**

**Figure S2**. Low IQ at 6 mo for both males and female infants who later received ASD diagnoses. The IQ (ELC) at 6 mo was significantly lower than the population mean of 100 for both males (p=2.9948e-11) and females (p=7.2749e-05); no difference in scores was detected between males and females (p>0.05).


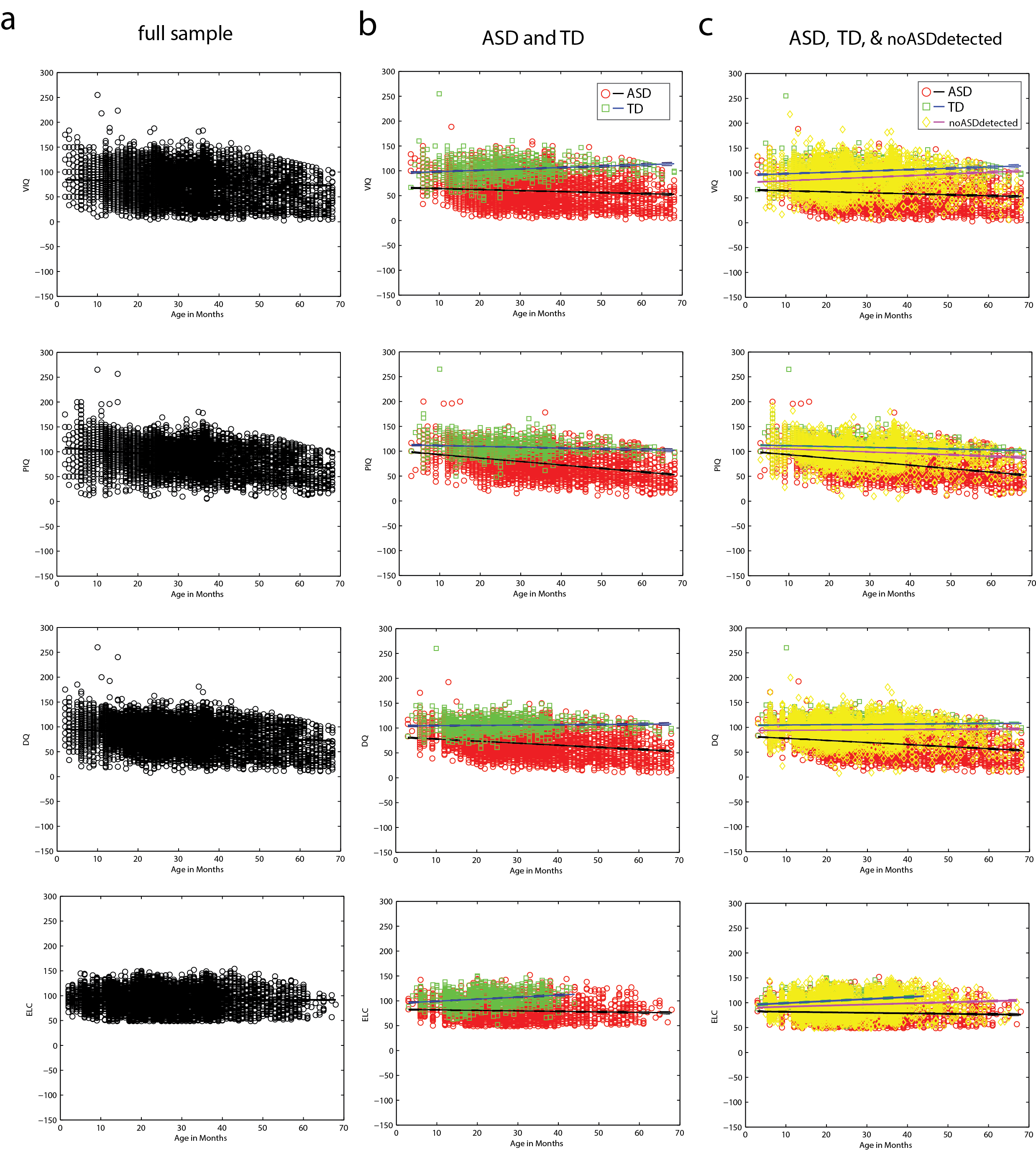


**Figure S3**. Linear multilevel models with age as predictor, for overall sample (a) and by subgroupings: ASD and TD are shown in (b), and ASD, TD, and ASD ruled out (‘noASDdetected’) are shown in (c). For all subgroupings, separate models were ran with response variables VIQ, PIQ, DQ, and ELC. Individual children were modeled as random effects.


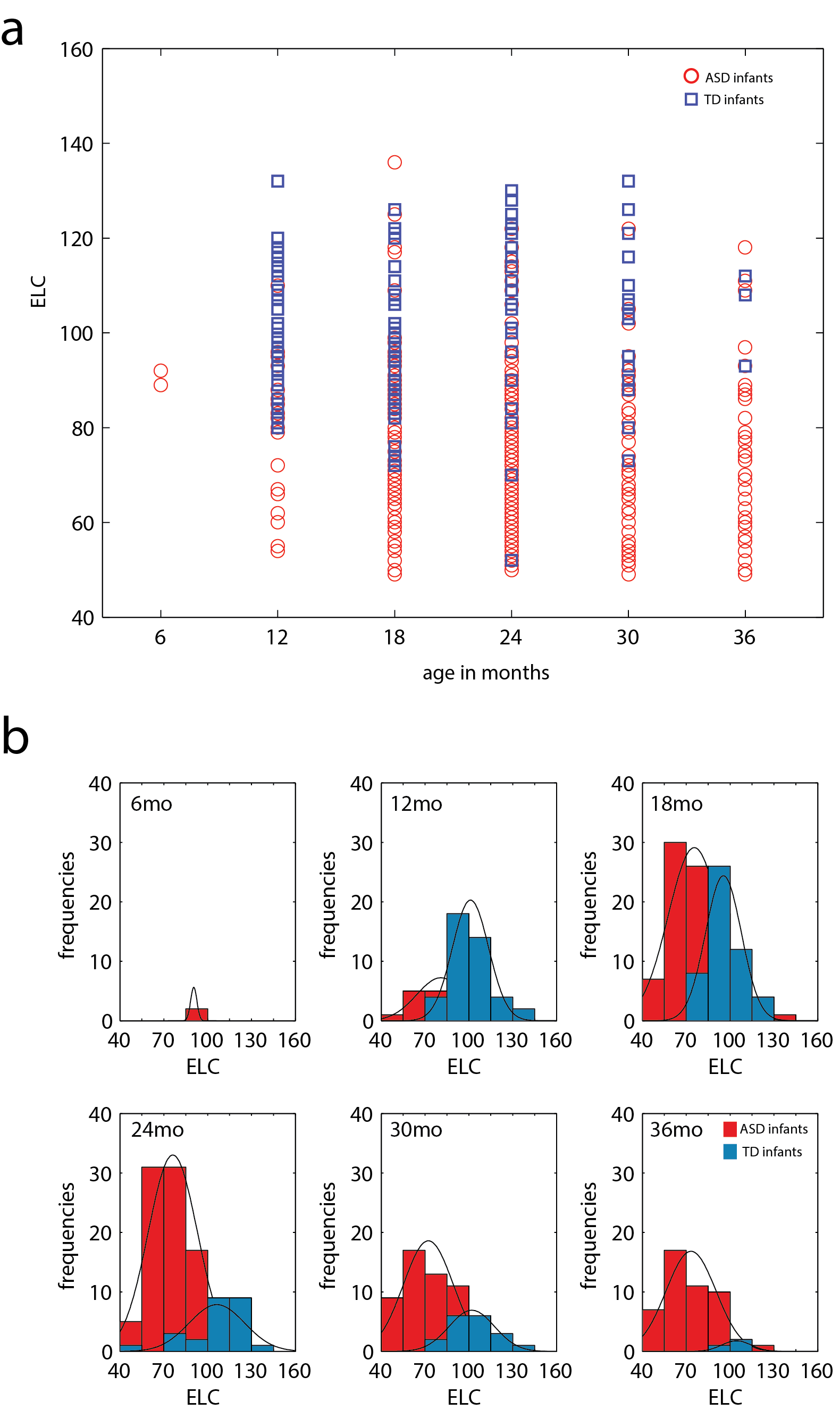


**Figure S4**. IQ by age groupings: age bins and frequency histograms for infants with ASD and TD outcomes, who have one and only one Mullen assessment falling into one of the pre-defined age bins. (a) Age bins (6, 12, 18, 24, 30, 36mo +/- 1 mo). (b) For each of the age bins in (a), frequency and probability density function (PDFs) are shown. The width of the bars is 15, equal to the standard deviation of the Early Learning Composite (ELC) standard score.


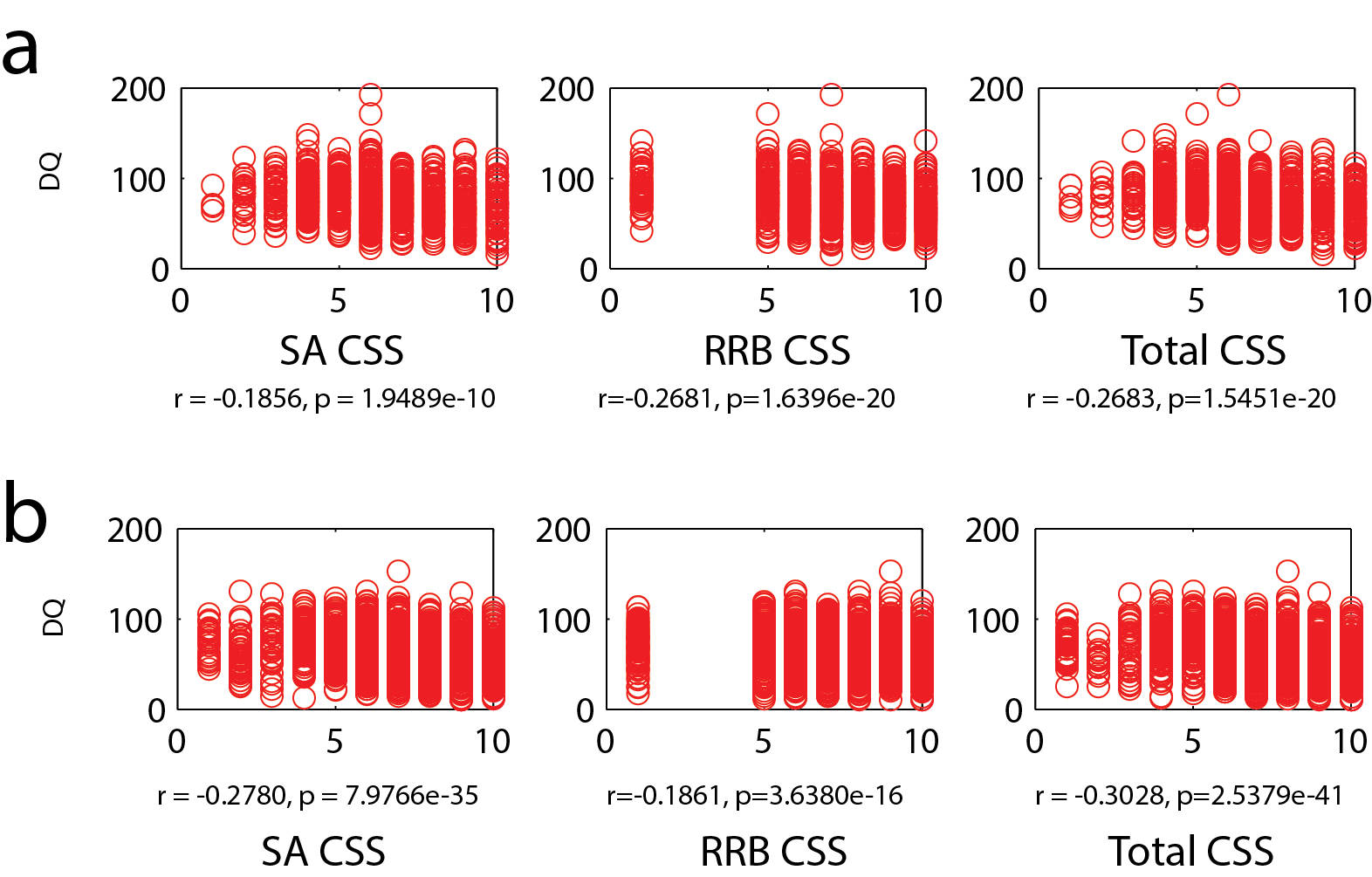


**Figure S5**. Lower IQ (Age-Equivalency-based DQ scores) significantly associates with worse autism symptoms. DQ vs Social Affect (SA), Repetitive and Restricted Behaviors (RRB) and Total Calibrated Severity Scores (CSS) on the ADOS. Same Ns as in Figure 4 in the main text: (a) from infants with at least 2 Mullen assessments and (b) from a separate set of infants with one and only 1 assessment on the Mullen who were ascertained with ASD. DQ: Developmental Quotient.
